# Nursing students' personality (Temperament and Character), burnout symptoms, and health and well-being

**DOI:** 10.1016/j.ijnsa.2024.100206

**Published:** 2024-05-06

**Authors:** Danilo Garcia, Maryam Kazemitabar, Elina Björk, Thiago Medeiros da Costa Daniele, Marko Mihailovic, Kevin M. Cloninger, Mirna Albuquerque Frota, C.Robert Cloninger

**Affiliations:** a Department of Behavioral Sciences and Learning, Linköping University, Linköping, Sweden; b Lab for Biopsychosocial Personality Research (BPS-PR), International Network for Well-Being; c Promotion of Health and Innovation (PHI) Lab, International Network for Well-Being; d Centre for Ethics, Law and Mental Health (CELAM), University of Gothenburg, Gothenburg, Sweden; e Department of Psychology, University of Gothenburg, Gothenburg, Sweden; f Yale School of Medicine, Yale University, New Haven, CT, USA; g Programa de Pós-graduação em Saúde Coletiva, University of Fortaleza (UNIFOR), Ceará, Brazil; h Anthropedia Foundation, St. Louis, Missouri, USA; i Department of Psychiatry, Washington University School of Medicine in St. Louis, St. Louis, Missouri, USA

**Keywords:** Health, Health promotion, Character, Nursing students, Person-oriented statistics, Personality, Temperament, Well-Being

## Abstract

**Background:**

About 9 million nurses will be needed by 2030. To face these unprecedented times, governments/institutions focus on educating as many nursing students as possible. This strategy is clouded by burnout and lack of both health and well-being among students and by the fact that personality is one of the major determinants of these health outcomes. Nevertheless, recent findings show that personality is a complex adaptive system (i,e., nonlinear) and that combinations of people's temperament and character traits (i.e., joint personality networks) might provide further information to understand its development, academic burnout, and lack of health and well-being.

**Aims:**

Our aims were to investigate the linear relationship between nursing students’ personality, burnout, health, and well-being; investigate the linear mediational effects of personality and burnout on health and well-being; and investigate differences in these health outcomes between/within students with distinct joint personality networks (i.e., nonlinear relationships).

**Method:**

Swedish nursing students (189 women, 29 men) responded to the Temperament and Character Inventory, The Maslach Burnout Inventory-General Survey for Students, and the Public Health Surveillance Well-Being Scale. We conducted correlation analyses and Structural Equation Modeling and, for the nonlinear relationships, Latent Profile Analysis and Latent Class Analysis for clustering and then Analyses of Variance for differences in health outcomes between/within students with distinct personality networks. This study was not pre-registered.

**Results:**

High levels of health and well-being and low burnout symptoms (low Emotional Exhaustion, low Cynicism, and high Academic Efficacy) were associated with low Harm Avoidance and high Self-Directedness. Some personality traits were associated with specific health outcomes (e.g., high Self-Transcendence-high Emotional Exhaustion and high Persistence-high Academic Efficacy) and their effects on health and well-being were mediated by specific burnout symptoms. Cynicism and Emotional Exhaustion predicted low levels of health and well-being, Academic Efficacy predicted high levels, and Cynicism lead both directly and indirectly to low levels of health and well-being through Emotional Exhaustion. We found two joint personality networks: students with an *Organized/Reliable* combination who reported being less emotionally exhausted by their studies, less cynical towards education, higher self-efficacy regarding their academic work/skills, and better health and well-being compared to nursing students with an *Emotional/Unreliable* combination.

**Conclusions:**

The coherence of temperament-character, rather than single traits, seems to determine students’ health outcomes. Thus, nursing education might need to focus on helping students to develop professional skills *and* health-related abilities (e.g., self-acceptance and spiritual-acceptance), by supporting self-awareness.

## Abstract (author highlights)

What is already known

• Since about 9 million nurses will be needed by 2030, governments and other institutions are focusing on educating as many nursing students as possible.
• There is an increasing prevalence of burnout and lack of both health and well-being among students.
• Personality is one of the major determinants of these health outcomes.

**What this** paper **adds**

• We show that approaching personality as a complex adaptive system (i,e., nonlinear) provides further information to understand its development, academic burnout, and lack of health and well-being.
• Specifically, the coherence of temperament-character dimensions, rather than single traits or single temperament and character profiles, seems to determine students’ health outcomes (i.e., burnout symptoms and health and well-being).
• Thus, nursing education might need to focus on helping students to develop professional skills and health-related abilities (e.g., self-acceptance and spiritual-acceptance), by supporting self-awareness.

## 1 Introduction

One of the major worldwide challenges of the 21st century is the high prevalence of burnout, mental illness, and lack of well-being among nursing professionals. This was further accentuated during the COVID-19 pandemic when nurses were, as always, on the frontlines—during this period, nurses had a significantly higher incidence of severe depression, psychological distress, and poor mental health compared to other helping professionals (Lai et al., 2020). To put the problem in perspective, the World Health Organization has estimated that about 9 million nurses will be needed by 2030 (WHO, 2020). Thus, to face these unprecedented times, there is a need to focus on training and educating as many nursing students as possible (Mihailovic et al., 2022; WHO, 2020). Nevertheless, this strategy is clouded by the fact that the increase of student burnout and ill-being are also major concerns in most countries (Erschens et al., 2019; Rudaz et al., 2023; Schaufeli et al., 2002; Tlili et al., 2021).

Nursing students’ responsibilities mirror, albeit to a lesser degree, the ones they will have as professionals, such as taking care of patients’ physiopsychosocial needs, providing emotional support and education to patients and their families, and working in highly competitive environments (Abudu-Birresborn et al., 2019; Chang et al., 2021; Labrague et al., 2018; Mathisen et al., 2022; Oliveira Silva et al., 2022; Suikkala et al., 2018; Sun et al., 2016; Usher et al., 2018). If that was not enough, as other students, nursing students also have academic concerns like class assignments, getting along with peers and professors, examinations, and both their own and others’ expectations regarding academic achievement. Some of the most common burnout symptoms among nursing students are feeling emotionally drained by their academic studies, having a cynical perspective on their education (e.g., doubting the significance of their education), and lacking confidence and self-efficacy in their academic ability and skills (Awa et al., 2010; Kamali et al., 2020). Even though academic burnout might result in multiple problems for nursing students, such as anxiety, academic failure, social dysfunction, and loss of motivation (Chae et al., 2020; Lee et al., 2017; Lyndon et al., 2017), the most prominent problem might be its effect on health and well-being. Indeed, the lack of life satisfaction and positive emotions in different health domains is more predictive of subsequent mortality and morbidity than the presence of negative emotions (Huppert and Whittington, 2003). In other words, we can expect that burnout symptoms might lead directly and indirectly to mental illness and ill-being. In this context, research among students show that both external and internal factors are associated with academic burnout (De la Fuente-Solana et al., 2019; Gómez-Urquiza et al., 2017; Molavynejad et al., 2019; Stoyanov, 2014; Stoyanov and Cloninger, 2012; Yang and Diefendorff, 2009). However, the role of individual differences in burnout has been neither well-studied nor reported (Cabras et al., 2023; Jacobs and Dodd, 2003; Mihailovic et al., 2022; Yanting M A, 2022).

### 1.1 Personality: a biopsychosocial system of motivation and adaptation

Since most of the few studies addressing this question often rely on lexical personality models (Solmi et al., 2020), which often regard personality as stable through life, we approached this endeavor using Cloninger's biopsychosocial model of personality. Cloninger's biopsychosocial model, as measured by the Temperament and Character Inventory (TCI), has been used in a plethora of studies since the 90′s (Garcia et al., 2017; Mihailovic et al., 2022). Moreover, the 20 different translations of the TCI have shown equal validity and reliability as the original English version (Garcia et al., 2017) and independent studies show that the TCI has equal or higher predictive validity compared with 11 of the most modern personality instruments (Grucza and Goldberg, 2007). Importantly, the main advantage of Cloninger's model is that it is not solely based on factor analytical approaches, as all lexical models are, but also on the solid theory of the evolution of the human brain—for critique on the use of factor analyses without theoretical basis see elswhere (Block, 1995; Gould, 1981; Proulx and Morey, 2021). As described in more detail in the Supplementary Material, distinctive brain regions represent unconscious and conscious processes in different memory systems that have been linked to specific neurotransmitters, brain networks, and forms of learning—temperament, which is responsible for emotional responses such as joy, sadness, anger, fear, disgust, and ambition; and character, which can be defined as individual differences in values, goals and self-conscious emotions (e.g., hope, love, and faith). In other words, temperament is responsible for our likes and dislikes and automatic emotional reactions, while character is what we make of ourselves intentionally (Cloninger, 2004). The distinction between these two personality domains (i.e., temperament and character) makes Cloninger's model suitable for describing and understanding individual differences between people but also within, thus allowing for understanding not only how people are different, as lexical models do, but also why people do what they do and how personality can be developed (Cervone, 2005, 2004).

Importantly, these systems of learning and memory can be dissociated functionally from one another, but normally interact jointly so that habits, goals, and values can be integrated (Cloninger, 2004; Cloninger and Cloninger, 2011; Garcia et al., 2019a, 2019c, 2019b). Indeed, in three recent molecular genetic studies (Zwir et al., 2021, 2020a, 2020b) using three culturally diverse samples (Finland, Germany, and South Korea), we found that the genes associated with personality do not only operate independently; but are organized as sets of particular clusters of genes. Thus, each gene can be expected to affect many traits and many genes affect each trait because evolutionary selection operates on whole organisms, not individual genes or traits (Zwir et al., 2022, 2021, 2020a, 2020b). In addition, as originally proposed by Cloninger, we found that the genes that encode variability in human temperament are relatively stable through life and are activated by stress reactivity and associative conditioning (i.e., the procedural brain system); while the genes encoding for human character not only support saliency detection, resolution of emotional conflicts, and social cooperation (i.e., the prepositional brain system), but also the emergence of human capacities for self-awareness, insight (i.e., immediate, accurate, and deep intuitive understanding), creative imagination, altruism, and autobiographical memory (i.e., the episodic brain system) (Zwir et al., 2022, 2021, 2020a, 2020b).

**Fig. 1:**
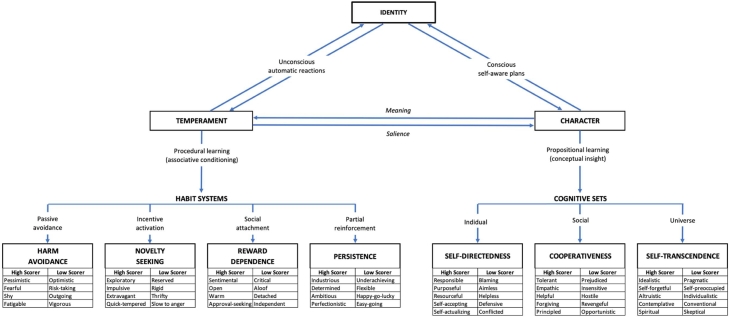
Cloninger's Biopsychosocial Model of Temperament and Character. Note. Reproduced with permission of the Anthropedia Foundation (www.anthropedia.org).

In sum, personality is a complex adaptive system of patterns of relationships between temperament structure, character structure, and histories of behavioral conditioning and insight (social) learning, that can be measured as a biopsychosocial model of personality (Fig. 1). Seeing personality as a dynamic complex adaptive system entails a nonlinear approach that is person-centered and in which an individual is not only adapting to the environment, but also to the traits within the person (Bergman et al., 2003; Bergman and Magnusson, 1997; Bergman and Wångby, 2014; Cloninger et al., 1997; Cloninger and Zwir, 2022, 2018)—that is, the notion of the individual as whole system unit which is best studied by analyzing profiles.

### 1.2 Joint personality (Temperament-Character) networks

Accordingly to the notion of personality as a complex adaptive system, recent research shows that there are three nearly separate joint personality networks of people with different combinations of temperament and character: *Creative/Reliable, Organized/Reliable, Emotional/Unreliable* (Zwir et al., 2021). There is marked complexity within each network in temperament-character relations. People in the Creative/Reliable network are low in Novelty Seeking (i.e., deliberate, thrifty, and orderly), low in Harm Avoidance (i.e., optimistic, confident, outgoing, and vigorous), high in Reward Dependence (i.e., sentimental, friendly, and approval seeking), and high in Persistence (i.e., determined) in combination with a mature character development (i.e., high in all three character traits), thus, leading to high levels of well-being and good health. Individuals in the Organized/Reliable network have the same temperament configurations as those in the Creative/Reliable network and are high in both Self-Directedness and Cooperativeness, but not in Self-Transcendence, which makes them healthy, but vulnerable to existential crises. Individuals in the Emotional/Unreliable network have the highest level of ill-being and the lowest level of well-being, which is logical since they are high in Novelty Seeking (i.e., impulsive and extravagant), high in Harm Avoidance (i.e., pessimistic, fearful, shy, and fatigable), and high in Reward Dependence (i.e., sentimental and friendly) in combination with an immature character development (i.e., low in all three character traits). This means that they may frequently have approach-avoidance conflicts, rejection sensitivity, disorganized attachments, and different biopsychosocial health issues. Individuals in the Creative/Reliable network have the highest levels of well-being, but also a slightly higher risk of ill-being than those in the Organized/Reliable network.

Importantly, some recencent cross-sectional replication studies of American, Portuguese, and Bulgarian populations (Garcia et al., 2022; Moreira et al., 2023, 2022, 2021) have shown that, consistent with extensive biopsychosocial evidence, women are more prosocial and healthier on average, particularly in Cooperativeness and Self-Transcendence. That being said, since character maturity develops with age; a Creative/Reliable joint personality network and high Self-Transcendence are relatively unlikely to be common in a young sample of nursing students. Nevertheless, traits as Cooperativeness and Reward Dependence should be expected as relatively high among nursing students, who are mostly women and have opted for a caring profession (Mihailovic et al., 2022).

### 1.3 Temperament and character, burnout, and health and well-being

Most past findings show that the temperament traits of Harm Avoidance and Persistence and the character trait of Self-Directedness are “*key traits*” for determining health care professionals and students’ susceptibility to psychopathology, burnout symptoms, resilience, well-being and even choice of profession and longevity (Chae et al., 2020; Eley et al., 2015; D. 2012, 2016, 2013; Kim et al., 2013; Lee et al., 2017; Stoyanov, 2014; Stoyanov and Cloninger, 2012). More specifically, self-directed behavior is a protective factor against psychopathology and promotes mental health and well-being, whereas executive harm avoidant behavior, such as pessimistic worrying and fear are synonymous with maladaptive emotions and behavior (Aldao et al., 2010; Ball et al., 2002; Chae et al., 2020, 2019; Izadpanah et al., 2016; Lee et al., 2023; Peirson and Heuchert, 2001). Persitence, on the other hand, is sometimes found to contribute to well-being and at other times to ill-being or not contribute to health outcomes at all when linear associations are investigated. Nevertheless, from nonlinear studies, it is clear that being persistent and industrious is self-defeating when the individual has difficulties in letting go or listening to body signals of exhaustion (i.e., low Self-Directedness), but that highly persistent behavior is helpful for self-actualization and well-being when the individual is optimistic, has the ability to let go of struggles, and is resourceful in finding new ways to approach problems (i.e., low Harm Avoidance and high Self-Directedness) (Cloninger, 2004; Cloninger et al., 2012; Granjard et al., 2021; Gusnard et al., 2003). In other words, these “*key traits*” need to be examined in relation to the joint combination of all temperament and character traits within the person. The lack of nonlinear research might also explain why some investigations among health care students with high prevalence of academic burnout, sometimes show that the tendency to be rigid and orderly (i.e., low Novelty Seeking) and unempathetic and mistrustful of others (i.e., low Cooperativeness) are predictive of academic burnout symptoms, such as feeling emotionally drained and cynicism toward one's education and lack of self-efficacy in one's academic work and ability (Khosravi, 2021).

## 2 The present study

The aim of the present study was to investigate the role of personality in nursing students’ burnout symptoms and health and well-being. More specifically, we 1) investigated the linear relationship between nursing students’ personality, burnout symptoms, and health and well-being, 2) investigated linear mediational effects by both personality traits and burnout symptoms on health and well-being, and for the nonlinear relationships we 3) investigated differences in burnout symptoms and health and well-being between and within nursing students with distinct joint personality networks.

## 3 Method

### 3.1 Ethical statement

Ethical approval was not required at the time the research was conducted as per national regulations. The study was performed in accordance with the ethical standards of the 1964 Helsinki declaration and its later amendments. The consent of the participants was obtained by virtue of survey completion after they were provided with all relevant information about the research, such as aims of the study, that participation was anonymous and voluntary, that they had the opportunity to ask questions, and that they were free to withdraw at any time without giving a reason and without cost or any repercussions regarding their education.

### 3.2 Participants and procedure

The data presented here was initially collected for a psychology thesis (Björk, 2021). First, a pilot study was conducted among five university students who did not find any problems or peculiarities regarding the survey's overall impression or with any part or individual items. After the pilot, the selection procedure for the actual study consisted of a convenience sample where program managers of nursing programs at 15 Swedish universities were asked to distribute the online questionnaire to their registered students. Seven program managers chose to allow their students to be asked to participate. The online survey was available for four weeks to all registered nursing students at the seven different universities. We only recruited participants among students who were registered for basic nursing programs, that is, nursing students in specialist education programs were not invited. A total of 354 respondents answered the survey. Regarding study form at the time of the survey, 46.90 % (*n* = 166) of the participants reported being engaged in their internship, 36.70 % (*n* = 130) stated regular studies, and 16.40 % (*n* = 58) did not answer to this question. Furthermore, 25.70 % of the participants reported being in year 1 of their studies, 34.20 % in year 2, and 33.50 % in year 3 (i.e., *n* = 325 and 29 missings). A total of 52 respondents were removed from the study due to incomplete responses or erroneous answers to validity questions. Of the remaining 302 participants, we had complete data (i.e., data used in the present study) for all the variables for 218 nursing students (189 women, 29 men) with a *mean* age of 28.46 years (*SD* = 8.44). This study was not pre-registered.

### 3.3 Measures

#### 3.3.1 Personality

The Temperament and Character Inventory (TCI) (Cloninger et al., 1993) was designed to measure individual differences in the four temperament dimensions and the three character dimensions of Cloninger's biopsychosocial model of personality (https://anthropedia.org). In the present study, we used the Swedish version comprising 60 items (TCI-3 60)—all 60 items are from the validated Swedish 240-item version (TCI-3) (Granjard et al., 2019). The items are answered in a 5-point Likert scale (1 = *Definitely False*; 5 = *Definitely True*), where a high score corresponds to high levels in each personality dimension: Novelty Seeking (e.g., “I often try new things just for fun or thrills, even if most people think it is a waste of time”), Harm Avoidance (e.g., “I often feel tense and worried in unfamiliar situations, even when others feel there is little to worry about”), Reward Dependence (e.g., “I like to discuss my experiences and feelings openly with friends instead of keeping them to myself”), Persistence (e.g., “I often push myself to the point of exhaustion or try to do more than I really can”), Self-Directedness (e.g., “In most situations my natural responses are based on good habits that I have developed”), Cooperativeness (e.g., “I often consider another person's feelings as much as my own”), and Self-Transcendence (e.g., “I sometimes feel so connected to nature that everything seems to be part of one living organism”). The TCI-3 60 also contains two validity questions (e.g., "Please click on 'Mostly or Probably True', this is a control question"). The *Cronbach's Alphas* for each of the personality dimensions in the present study were as follows: Harm Avoidance = 0.77, Novelty Seeking = 0.63, Reward Dependence = 0.62, Persistence = 0.70, Self-Directedness = 0.68, Cooperativeness = 0.67, and Self-Transcendence = 0.74.

#### 3.3.2 Burnout

The Maslach Burnout Inventory - General Survey for Students (Maslach et al., 1996) was translated from English to Swedish and then back-translated with permission from MindGarden, Inc. (https://www.mindgarden.com). The validation of the translation was partially based on the Swedish version of the Maslach Burnout Inventory - General Survey (Hallberg and Sverke, 2004) since both versions are relatively similar. For clarity reasons, we did minor reformulations of statements where the word ‘work’ was replaced by ‘studies’. Furthermore, since the current study was conducted during the Covid-19 pandemic in which most classes were conducted online rather than on campus, we found it appropriate to change some items that implied campus-based studies. For example, the item "I feel used up at the end of the day at the university" was reformulated as "I feel used up after a day of studies". The inventory consists of 16 items to which the participant estimates how often they experience what is postulated in each item using a 7-point Likert scale (0 = *Never*, 6 = *Every Day*). A higher score implies a higher level of Emotional Exhaustion (e.g., “I feel emotionally drained by my studies”), Cynicism (e.g., "I doubt the significance of my studies"), and Academic Efficacy (e.g., "While studying, I feel confident that I am effective at getting things done."). Accordingly, burnout is then indicated by high scores in Emotional Exhaustion and Cynicism and a low score in Academic Efficacy. The version used here had the following *Cronbach's Alphas*: Emotional Exhaustion = 0.91, Cynicism = 0.84, and Academic Efficacy = 0.83.

#### 3.3.3 Health and well-being

The Public Health Surveillance Well-Being Scale (Bann et al., 2012) consists of a total of ten statements that aim to measure health and well-being based on mental, physical, and social components (i.e., biopsychosocial health). The scale was translated from English to Swedish and then back-translated with permission from the original authors. The instrument is based on several rating scales with different response levels where the participant is asked for their degree of satisfaction, purpose, and accomplishment with life in general (“I am satisfied with my life”, “My life has a clear sense of purpose”, “Most days I feel a sense of accomplishment from what I do”; 1 = *Strongly Disagree*, 5 = *Strongly Agree*) and satisfaction with different life domains, such as family life, friends and social life, and energy level (1 = *Very Dissatisfied*, 10 = *Very Satisfied*). The participant is also asked to report the frequency of feeling “cheerful” and “hopeless” for the last 30 days (1 = *None of the Time*, 5 = *All of the Time*), to estimate their individual experience of general health ("In general, would you say your health is…? "; 1 = *Excellent*, 5 = *Poor*), and to report their daily experience of feeling “very healthy and full of energy" during the last 30 days (*Number of Days* = Free Text Response, 1 = *None/Zero Days*, 2 = *Don't Know/Not Sure*). The different response levels were coded (or recoded for the question on general health) and standardized as recommended by Bann et al. (2012), where a higher score indicates a higher level of good biopsychosocial health and well-being. In the present study, *Cronbach's Alpha* for this scale was 0.87.

### 3.4 Statistical treatment

All analyses were conducted using SPSS version 26, Amos version 24, and Mplus version 8.3. The normality of the data was checked and approved by investigating skewness and kurtosis—all values were between ±1.96. For the instruments measuring personality and burnout, a maximum of 5 % internal missing data was found acceptable for considering respondents’ answers as valid (Cloninger et al., 2019; Granjard et al., 2019). However, for the health and well-being measure, 10 % internal missing was considered acceptable (Bann et al., 2012). The missing items for each individual dimension in each measure were replaced with the mean value of the participant's answers in that dimension (Pigott, 2009).

After controlling for normality of the data and handling the missing data, we conducted our first main set of analyses to investigate the linear relationships between the nursing students’ self-reported personality, burnout symptoms, and health and well-being: a correlation analysis and a structural equation modeling (SEM) in which we also investigated mediational paths. Before our second main analyses, we first used Latent Profile Analysis (LPA) to identify temperament profiles and character profiles, separately, within the population of nursing students (see Supplementary Material for the details). LPA helped us to determine a set of mutually distinct latent temperament and character profiles of nursing students by clustering individuals with similar temperaments, respectively, character responses. In other words, the latent temperament and character profiles are subgroups of individuals with similar characteristics on a specific latent construct (Coyle et al., 2019; Garcia et al., 2023b). After the profiling, as part of the nonlinear analyses and before testing differences in burnout symptoms and health and well-being, we applied Latent Class Analysis (LCA) to the nursing students’ temperament profiles and character profiles to create the joint personality networks. LCA is a person-centered mixture modeling that categorizes latent profiles within the sample derived from patterns of responses to observed variables (Muthen and Muthen, 2000). In short, through LCA, we clustered the LPA-generated temperament profiles and character profiles into distinct joint profiles representing both personality dimensions as networks. In other words, nursing students with similar temperament profiles and character profiles were assigned to separate joint personality networks. After profiling the nursing students in distinct joint personality networks, we standardized participants’ scores in burnout symptoms and health and well-being— since these measures had different rating scales. Then, we investigated differences in burnout symptoms and health and well-being using Multiple Analyses of Variance (MANOVA) between nursing students with distinct joint personality networks.

That is, in contrats to the first main analyses using correlations and SEM (i.e., linear analyses), calculating the joint personality networks allowed us to investigate the relationship between personality and burnout symptoms and health and well-being in a nonlinear manner.

## 4 Results

### 4.1 Correlation between personality, burnout symptoms, and health and well-being

Table 1 shows the Pearson correlations between personality, burnout symptoms, and health and well-being as well as means and standard deviations for all variables. As expected, Harm Avoidance was negatively related to health and well-being and Academic Efficacy and positively related to Emotional Exhaustion and Cynicism. Also, as expected, Self-Directedness showed exactly the opposite pattern; it was positively related to health and well-being and Academic Efficacy and negatively related to Emotional Exhaustion and Cynicism. Persistence followed the same pattern as Self-Directedness, with the exception of the relationship to Emotional Exhaustion, which was non-significant. The only other trait that showed significant correlations was Reward Dependence, which was positively related to Academic Efficacy and health and well-being. Last but not least, self-reported health and well-being were negatively associated with Emotional Exhaustion and Cynicism and positively to Academic Efficacy.

**Table 1:** Mean and standard deviation and correlations between burnout symptoms, health and well-being, and personality traits.

Note. SD: standard deviation. Colored cells marking correlations above 0.20 (i.e., the recommended minimum effect size representing a practical significant effect for social science data according to Ferguson, 2009) between personality traits and burnout symptoms (blue), personality traits and health and well-being (yellow), and health and well-being and burnout symptoms (green).

Note: *N* = 218.

### 4.2 Structural equation modeling (SEM)

**Fig. 2:**
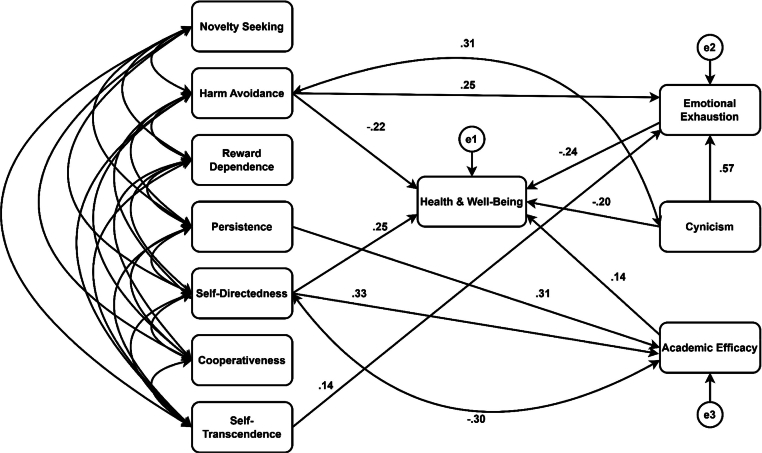
Standardized regression weights and correlations between personality traits, burnout symptoms, and health and well-being among nursing students. Note: *N* = 218.

After seven iterations, we obtained a model (*χ^2^/df* = 2.39, *p* < .001, *GFI* = 0.96, *CFI* = 0.95, *RMSEA* = 0.08) that we considered had very good fit to the data (Hu and Bentler, 1999). We bootstrapped data with 95 % bias-corrected confidence interval in order to acquire more accurate results for mediation effects in the model. Fig. 2 depicts the standardized regression weights and correlations between personality dimensions, burnout symptoms, and health and well-being.

Regarding personality traits and health and well-being, Harm Avoidance had a negative effect (*β* = −0.22, *p* < .05) and Self-Directedness a positive effect on health and well-being (*β* = 0.25, *p* < .05). Regarding burnout symptoms and health and well-being, Emotional Exhaustion (*β* = −0.24, *p* < .05) and Cynicism had a negative effect (*β* = −0.20, *p* < .01) and Academic Efficacy had a positive effect on health and well-being (*β* = 0.14, *p* < .05). Regarding the relationship between personality dimensions and burnout symptoms, Harm Avoidance (*β* = 0.25, *p* < .05) and Self-Transcendence (*β* = 0.14, *p* < .05) had a positive effect on Emotional Exhaustion, Persistence (*β* = 0.31, *p* < .05) and Self-Directedness (*β* = 0.33, *p* < .05) had a positive effect on Academic Efficacy. Moreover, Harm Avoidance had a positive effect (*β* = 0.31, *p* < .01) and Self-Directedness had a negative effect (*β* = −0.30, *p* < .01) on Cynicism. Finally, Cynicism had a strong positive effect on Emotional Exhaustion (*β*=0.57, *p* < .05).

Additionally, the model also showed that some personality dimensions and burnout symptoms had indirect effects on health and well-being. Self-Transcendence had a small indirect negative effect on health and well-being (*β* = −0.03, *p* < .01) through Emotional Exhaustion as the mediator. Persistence had a small indirect positive effect on health and well-being (*β* = 0.04, *p* < .01) that was mediated by Academic Efficacy. Harm Avoidance had an indirect negative effect on health and well-being through Emotional Exhaustion as the mediator (*β* = −0.06, *p* < .01), hence, the total effect of Harm Avoidance on health and well-being was larger (*β* = −0.28, *p* < .01). Self-Directedness had an indirect positive effect on health and well-being through Academic Efficacy as the mediator (*β* = 0.05, *p* < .01), hence, the total effect of Self-Directedness on health and well-being increased to *β* = 0.30, *p* < .01. Finally, Cynicism had an indirect negative effect (*β* = −0.13, *p* < .05) on health and well-being through Emotional Exhaustion as the mediator, hence, the total negative effect of Cynicism on health and well-being was much higher (*β* = −0.33, *p* < .05).

In sum, the linear analyses showed that high levels of health and well-being and low levels in all burnout symptoms (low Emotional Exhaution, low Cynicism, and high Academic Efficay) were associated to low Harm Avoidance and high Self-Directedness. Nevertheless, some single traits were also associated to specific health outcomes, such as, high Self-Transcendence to high Emotional Exhaustion and high Persistence to high Academic Efficacy. The effects of these personality dimensions on health and well-being were mediated by specific burnout symptoms. Lastly, the burnout symptoms of Cynicism and Emotional Exhaustion predicted low levels of health and well-being, while Academic Efficacy predicted high levels. Cynicism per se leads to high levels of Emotional Exhaustion, thus leading both directly and indirectly to low levels of health and well-being.

### 4.3 Latent class analysis (LCA): joint personality (Temperament-Character) networks

As mentioned in the Statistical Treatment section, before conducting the LCA, we calculated the temperament profiles and character profiles for each nursing student separately using Latent Profile Analysis (LPA). The LPA revealed three temperament profiles and three character profiles. Next, we only recapitulate the results and derived conclusions to make the results of the Joint Personality (Temperament-Character) Networks more clear for the reader. For the details, please see Supplementary Materials.

Regarding the temperament profiles, nursing students with temperament profile 1 have much social warmth due to high Reward Dependence (R) and are likely to be careful and dutiful in carrying out responsibilities assigned to them due to high Novelty Seeking (n); they avoid doing anything that exposes them to risk of danger, rejection, criticism due to high Harm Avoidance and high Reward Dependence (HR), and avoid also testing new ways of doing things due to low Novelty Seeking (n) and have difficulty initiating anything new because of their inhibitions rooted in their tendency to pragmatism and underachievement (low Persistence = *p*) and their fears of rejection, criticism, loss, and change due to high Harm Avoidance and high Reward Dependence (HR). Hence, we labeled this temperament profile (*n* = 44, 20.2 % of the nursing students) as *pessimistic (nHRp)*. The nursing students in temperament profile 3 were also low in Novelty Seeking (n), high in Harm Avoidance (H), high in Reward Dependence (R), but high in Persistence (P). Hence, we labeled this temperament profile (*n* = 17, 7.2 % of the nursing students) as *painstaking (nHRP)* because individuals with this profile are both anxious (nHR) and determined (P). Despite the similarities of temperament profiles 1 and 3, that is, both being cautious (nHR); nursing students in the painstaking (nHRP) temperament profile were extremely more persistent (P) and showed less differences within temperament dimensions, thus, the tendencies to being inhibited (nH), rejection-sensitive (HR), and traditional (nR) are more accentuated among nursing students in this temperament profile. The nursing students in temperament profile 2 (*n* = 157, 72 % of the nursing students) might be described as *reliable (nhRP)* because they were low in Novelty Seeking (n), low in Harm Avoidance (h), high in Reward Dependence (R), and high in Persistence (P). Nursing students with this temperament profile are stable (nh), warmly sociable (hR), traditional (nR), and hard-working (P), thus, they carry out what they are expected to do in a predictable and traditional manner and usually develop a mature character (see Supplementary Materials for the details).

Regarding the character profiles, character profile 1 was labeled *apathetic (sct)* because the nursing students in this character profile (*n* = 21, 9.6 % of the nursing students) reported low Self-Directedness (s), low Cooperativeness (c), and low Self-Transcendence (t). In other words, they tend to feel victimized and helpless (sc), show very poor judgment (st) and are distrustful (ct), thus, they experience the world from an outlook of separateness, which leads to fear, excessive desire, and false pride or self-reproach. The nursing students in character profile 2 (*n* = 130, 59.6 % of the nursing students) reported high levels of Self-Directedness (S), high levels of Cooperativeness (C), and low levels of Self-Transcendence (t) and might be described as *organized (SCt)*. In other words, they tend to be perceived as mature leaders (SC), logical (St), and conventional (Ct), but when they face difficult existential challenges, such as severe illness or death, they often lack the necessary outlook of unity and connectedness needed to be resilient through such situations (t). Lastly, the nursing students in character profile 3 (*n* = 67, 30.7 % of the nursing students) were high in all three character dimensions and might therefore be described as *creative (SCT)*. Individuals with a creative (SCT) profile tend to be constructive, keep things in perspective when faced with challenges (S), enjoy helping others, are compassionate (C), and seek to grow in awareness of things that go beyond human existence (T).

The LCA for the joint personality networks resulted in different models with distinct numbers of profiles. We tested the fit indices for four joint personality network models (see Supplementary Material) and found that Model 2, with two networks, had the lowest AIC, BIC, and SABIC and the highest entropy (see Table 2). The most common temperament-character combination in joint personality network 1 (*n* = 68, 31.2 % of the nursing students) was a student with a pessimistic (nHRp) temperament profile with an organized (SCt) character profile—about 32.4 % of the nursing students in this joint personality network had this specific temperament and character combination (see Table 3). In joint personality networks 2 (*n* = 150, 68.8 % of the nursing students), the most common combination was the reliable (nhRP) temperament profile and the organized (SCt) character profile, with about 58.7 % of the nursing students within joint personality network 2 having this combination (see Table 4). More importantly, within joint personality network 1, 83 % of the students with an organized (SCt) character profile and 81 % of the students with a creative (SCT) character profile had a dysfunctional temperament profile, that is, either pessimistic (nHRp) or painstaking (nHRP). Furthermore, only 1 out of the 11 (about 9 %) nursing students with an apathetic (sct) character profile had a reliable (nhRP) temperament profile (see Table 3). On the other hand, within joint personality network 2 (Table 4), only 1 % of the students with an organized (SCt) character profile and 2 % of the students with a creative (SCT) character profile had a pessimistic (nHRp) temperament profile, while 8 out of the 10 (about 80 %) nursing students with an apathetic (sct) character profile had a reliable (nhRP) temperament profile.

**Table 2:** Fit indices for the four joint personality (temperament-character) network models extracted using Latent Class Analysis (LCA).

Note. The number of the model also indicates the number of networks in that specific model. Green indicates the chosen model.

**Table 3:** Crosstabulation of the temperament profiles and character profiles in joint personality networks 1 (Emotional/Unreliable).

			**Character Profiles**			**Total**
			**Apathetic (sct)**	**Organized (SCt)**	**Creative (SCT)**	
**Temperament Profiles**	**Pessimistic (nHRp)**	Count	10	22	8	40
		% within Character Profile	90.9 %	53.7 %	50.0 %	58.8 %
		% of Total	14.7 %	32.4 %	11.8 %	58.8 %
	**Reliable (nhRP)**	Count	1	7	3	11
		% within Character Profile	9.1 %	17.1 %	18.8 %	16.2 %
		% of Total	1.5 %	10.3 %	4.4 %	16.2 %
	**Painstaking (nHRP)**	Count	0	12	5	17
		% within Character Profile	0.0 %	29.3 %	31.3 %	25.0 %
		% of Total	0.0 %	17.6 %	7.4 %	25.0 %
**Total**		Count	11	41	16	68
		% within Character Profile	100.0 %	100.0 %	100.0 %	100.0 %
		% of Total	16.2 %	60.3 %	23.5 %	100.0 %

Note. *n* = low Novelty Seeking, *H* = high Harm Avoidance, *h* = low Harm Avoidance, *R* = high Reward Dependence, *P* = high Persistence, *p* = low persistence, *S* = high Self-Directedness, *s* = low self-directedness, C = high Cooperativeness, *c* = low cooperativeness, *T* = high Self-Transcendence, *t* = low self-transcendence.

**Table 4:** Crosstabulation of the temperament profiles and character profiles in joint personality networks 2 (Organized/Reliable).

			**Character Profiles**			**Total**
			**Apathetic (sct)**	**Organized (SCt)**	**Creative (SCT)**	
**Temperament Profiles**	**Pessimistic (nHRp)**	Count	2	1	1	4
		% within Character Profile	20.0 %	1.1 %	2.0 %	2.7 %
		% of Total	1.3 %	0.7 %	0.7 %	2.7 %
	**Reliable (nhRP)**	Count	8	88	50	146
		% within Character Profile	80.0 %	98.9 %	98.0 %	97.3 %
		% of Total	5.3 %	58.7 %	33.3 %	97.3 %
**Total**		Count	10	89	51	150
		% within Character Profile	100.0 %	100.0 %	100.0 %	100.0 %
		% of Total	6.7 %	59.3 %	34.0 %	100.0 %

Note. *n* = low Novelty Seeking, *H* = high Harm Avoidance, *h* = low Harm Avoidance, *R* = high Reward Dependence, *P* = high Persistence, *p* = low persistence, *S* = high Self-Directedness, *s* = low self-directedness, C = high Cooperativeness, *c* = low cooperativeness, *T* = high Self-Transcendence, *t* = low self-transcendence.

Last but not least, using a one-way MANOVA, we found significant differences in temperament and character dimensions between the two joint personality networks. Compared to nursing students with the joint personality network 2, nursing students in joint personality network 1 scored significantly lower in Novelty Seeking, significantly higher in Harm Avoidance, significantly lower in Persistence, significantly lower in Self-Directedness, equally high in Reward Dependence and Cooperativeness, and equally low in Self-Transcendence. Thus, suggesting that nursing students in joint personality network 1 had an *Emotional/Unreliable* personality combination and that those in network 2 had an *Organized/Reliable* combination. See Supplementary Material for the details (Figure S8).

### 4.4 Differences in burnout symptoms and health and well-being between and within nursing students with distinct joint personality (Temperament-Character) networks

**Fig. 3:**
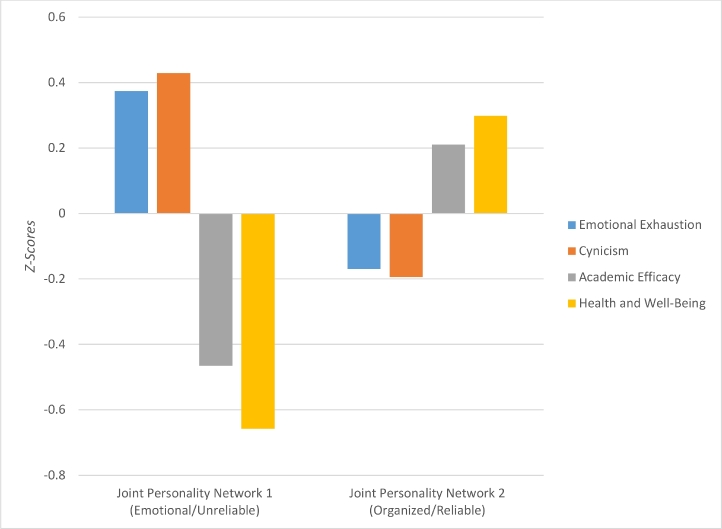
Mean (*z-scores*) differences in burnout symptoms and health and well-being between and within joint personality networks. Note: Emotional/Unreliable *n* = 68; Organized/Reliable *n* = 150.

Using a one-way MANOVA, we found significant differences in burnout symptoms and health and well-being between nursing students with distinctive joint personality networks (Wilks’ Lambda = 0.28, F_(4, 213)_ = 14.58, *p* < .001, partial Eta squared = 0.22). Pairwise comparison, using a Bonferroni correction to the alpha level, indicated that all differences were significant (*p* < .001). Fig. 3 displays the differences in burnout symptoms and health and well-being between the joint personality networks. In short, nursing students in joint personality network 2 (Organized/Reliable) reported better health and well-being, were less emotionally exhausted, less cynical towards their academic education, and felt higher self-efficacy with regard to their study work and skills.

Using one repeated measures ANOVA for each joint personality network, we investigated differences within individuals with regard to burnout symptoms and health and well-being. For the nursing students in joint personality network 1 (Emotional/Unreliable), the test of within-subject effects with Greenhouse-Geisser correction was significant (F_(1.13, 2755.87)_ = 239.46, *p* < .001, partial Eta squared = 0.78). The post hoc test with Bonferroni correction showed that all mean differences within this network were significant except for that between Emotional Exhaustion and Professional Efficacy and between Cynicism and Professional Efficacy (*p* > .05). In other words, nursing students within joint personality network 1 (Emotional/Unreliable), had lower levels of health than burnout symptoms, higher levels of Cynicism than Emotional Exhaustion, and they had as low levels of Professional Efficacy as their high levels of Emotional Exhaustion and Cynicism. The test within-subjects effect for joint personality network 2 (Organized/Reliable) with Greenhouse-Geisser correction indicated significant differences as well (F_(1.11, 6382.91)_ = 1213.19, *p* < .001, partial Eta squared = 0.89). The post hoc test showed that all mean differences within this network were significant (*p* < .01). In other words, nursing students within joint personality network 2 (Organized/Reliable), had higher levels of health than burnout symptoms but higher levels of Professional Efficacy than both Cynicism and Emotional Exhaustion, and they had lower levels of Emotional Exhaustion than any of the other burnout symptoms (see Fig. 3).

## 5 Discussion

The aims of the present study were to 1) investigate the linear relationship between nursing students’ personality, burnout symptoms, and health and well-being, 2) investigate linear mediational effects of both personality traits and burnout symptoms on health and well-being, and for the nonlinear analyses we 3) investigated differences in burnout symptoms and health and well-being between and within nursing students with distinct joint personality networks.

In essence, the linear analyses showed that high levels of health and well-being and low levels of burnout symptoms (i.e., low Emotional Exhaustion, low Cynicism, and high Academic Efficay) were associated with low Harm Avoidance and high Self-Directedness. This was expected, since similar results have been found among medical students, doctors, newly graduated nurses, and other health care professionals (Eley et al., 2016, 2013; Lee et al., 2017; Mihailovic et al., 2022)—that is, resilience, well-being, and adaptive self-regulation is predicted by the tendency of being optimistic rather than pessimistic, outgoing rather than shy, energetic, resourceful, self-acceptant, responsible rather than blaming, having good habits that are in coordance to one's goals, and having purpose with one´s life. Nevertheless, some single traits were also associated to specific health outcomes, such as high Self-Transcendence to high Emotional Exhaustion, which in turn lead to low levels of health and well-being; and high Persistence to high Academic Efficacy, which in turn lead to high levels of health and well-being.

Hence, nursing students who frequently experience flow, identify with nature and the world around, have highly humanistic values, and accept the mysteries of life (i.e., hight Self-Transcendence) have a tendency to also feel drained by their academic work. In this context, is noteworthy to mention that Swedes along other Scandinavians seem to not value spirituality, but rather value scepticism, pragmatism, and conventionalism (Josefsson et al., 2013). Indeed, also anthropological and social observations indicate that spirituality is frown upon in sweden (Thurfjell, 2015), which might explain why high Self-Transcendence is detrimental for health among nursing students and other Swedish sub-populations, such as adolescents (Schültz, 2015; Schütz et al., 2013). Additionally, nursing students who are high in Self-transcendence might experience flow and feel connected to their patients at a humanistc level, which might be expressed as loosing track of time and high emotional engagement when they are studing; thereby to exhaustion, specially if the students are also high in Persistence and/or if they have problems self-regulating those emotions. For instance, individuals who are low in Self-directedness but high in both Cooperativeness and Self-Transcendence have moods that fluctuate up and down because they are trustful due to high Cooperativeness and high Self-Transcendence, but they are also submissive due to low Self-Directedness and high Cooperativeness and illogical due to low Self-Directedness and high Self-Transcendence (Cloninger, 2004). Interestingly, our results showed that nursing students who are industrious, determined, and ambitious feel confident about their academic skills and self-efficacy. This is in line with studies relating such type of behavior, measured using the TCI and other personality models, to academic success and performance at work (Mihailovic et al., 2022; Ozer and Benet-Martínez, 2006; Roberts et al., 2007; Roberts and Hogan, 2001; Soto, 2019; Soto et al., 2021; Soto and Tackett, 2015). Nevertheless, as we will discuss later, highly persistent individuals are also perfectionistic and have difficulties letting go or listen to signs of exhaustion, which, migh lead to burnout in some individuals (Cloninger et al., 2012; Garcia et al., 2012; Gusnard et al., 2003). Likewise, Self-Transcendence is needed in order to adapt wisely to life challenges, specially when we get older or when we face existential issues like nursing students will do later in both their private and work life (Cloninger, 2013; C.R. 2012, 2007, 2006).

Also as expected, the burnout symptoms of Cynicism and Emotional Exhaustion predicted low levels of health and well-being, while Academic Efficacy predicted high levels (Chae et al., 2020; Lee et al., 2017; Lyndon et al., 2017; Stoyanov, 2014; Stoyanov and Cloninger, 2012). Moreover, high Cynicism per se lead to high levels of Emotional Exhaustion, thus leading both directly and indirectly to low levels of health and well-being. In other words, humor and jargon involving sarcasm might be detrimental to the health of students who aspire becoming helping professionals. That being said, humor is a multifaceted characteristic that under specific conditions (e.g., using light humor to help patients cope) might be helpful and even important to integrate in health care educational situations (Raecke and Proyer, 2022), but a cynical view is detrimental to health and other important outcomes. For instance, a study among nursing staff indicated that higher levels of Cynicism increased their levels of Emotional Exhaustion, in turn, leading to decreased job performance and even decreased care quality (Manzano García and Ayala Calvo, 2012). These results verify that academic burnout has a negative effect on nursing students’ health and well-being in addition to leading to mental health problems, such as, anxiety, academic failure, social dysfunction, and loss of motivation (Chae et al., 2020; Lee et al., 2017; Lyndon et al., 2017),

The nonlinear analyses revealed two joint personality networks. Nursing students with an Organized/Reliable personality combination, compared to nursing students with an Emotional/Unreliable personality combination, reported being less emotionally exhausted by their academic work, less cynical towards their education, higher self-efficacy regarding their academic work and skills, and better health and well-being in different life domains. First, it is important to point out that, as expected, we did not found a Creative/Reliable network in this sample of nursing students. We expected the Creative/Reliable joint personality network and high Self-Transcendence to be unlikely in a young sample of nursing students because character maturity develops with age (Josefsson et al., 2013). Also as expected, Cooperativeness and Reward Dependence were relatively high in this sample of nursing students, who are mostly women and have opted for a caring profession (Mihailovic et al., 2022).

Second, the analyses of the joint personality networks showed that the association of high Persistence and less burnout symptoms and better health and well-being might not be as straightforward as the linear analyses showed. Indeed, while about 58.8 % of the nursing students in the Emotional/Unreliable network were low in Persistence, that is had a pessimistic (nHRp) temperament profile, the rest (41.2%) were high in Persistence with either a painstaking (nHRP) or a reliable (nhRP) temperament profile. In other words, suggesting that high Persistence is not good for health if is combined with inadaptive self-regulation/low character maturity (Cloninger et al., 2012; Garcia et al., 2012). Also in this line, the nonlinear analyses showed that the tendency to be rigid rather than impulsive and orderly rather than disorderly (i.e., low Novelty Seeking vs. high Novelty Seeking), as all nursing students in the present study were, might be adaptive if the individual has an Organized (SCt) or a Creative (SCT) character profile. Thus, explaining why some linear studies among health care students with high prevalence of academic burnout, sometimes show that low Novelty Seeking is predictive of academic burnout symptoms (Khosravi, 2021). Therefore, we suggest that is rather the whole personality combination that is responsible for positive or negative outcomes. A single “positive” trait, a stable temperament profile, or not even a creative (SCT) character profile separetly seems to be sufficient for optimal health and well-being. Is rather the coherence of temperament and character that determines positive health outcomes (Cloninger, 2004).

### 5.1 Limitations and strengths

Our study sample was limited to nursing students and relatively small. This was probably a result of the survey being distributed online rather than in person—online surveys have been shown to lead to higher drop out due to participants experiencing, for example, lower interest and higher burden (Stieger et al., 2007). Thus, we need to replicate the results in larger populations of nursing students and probably among different cultures. That being said, as shown by the World Value Survey (Inglehart, 2018a, Inglehart, 2018b), Sweden is at the top of both secular-rational values (i.e., emotionally stable, pragmatic, liberal) and self-expressive values (i.e., pro-social, open, and idealistic). The personal characteristics behind secular-rational values are typical of individuals who are low in Novelty Seeking and high in Reward Dependence (i.e., traditional), whereas being self-determined (i.e., high Self-Directedness), helpful, empathetic, and prosocial (i.e., high in Cooperativeness) is typical of individuals with self-expressive values. In addition, as discussed earlier, Swedes report decreases in spirituality, such as becoming more sceptical, pragmatic, and conventional (i.e., low Self-Transcendence), with increasing age (Josefsson et al., 2013), which is usually associated with high negative emotions (Cloninger, 2004). Indeed, high spirituality is somewhat frown upon in Sweden (Thurfjell, 2015), which might explain why it is associated with high ill-being among different Swedish populations (Schültz, 2015). Hence, despite the sample not being representative, the tempermament profiles, the character profiles, and the joint personality networks mirror relatively well the results from the World Value Survey with regards to Sweden.

### 5.2 Conclusion and further remarks

Untreated burnout is likely to become resistant to interventions and end up in, besides depression and anxiety, psychosomatic problems, musculoskeletal pain, and cardiovascular diseases (Stoyanov, 2014; Stoyanov and Cloninger, 2012). The results of the current study might help stakeholders, policymakers, and researchers to address academic burnout among nursing students because they point out the need to focus on health promotion through the support of personal development and a supportive study climate (Stoyanov, 2014; Stoyanov and Cloninger, 2012). Such activities might, for example, involve pedagogical health promotion strategies (Bispo et al., 2023) and well-being interventions. Our research network has for example conducted psychoeducative coaching interventions that include recovery and stress-reducing activities that show positive and promising results among students and other populations by promoting positive development of the human condition, that is, higher levels of Self-Directedness, Cooperativeness, and Self-Transcendence (Cloninger and Cloninger, 2011; Garcia et al., 2023a, 2019a, n.d.) —see also (del Val-et al., 2024). In this endavor we need to take into consideration that university teachers with less than eleven years of teaching experience, for example, report lower levels of self-esteem compared to older teachers with more experience (Capelo et al., 2022). Thus, to achieve student health and resilience, universities need to also promote personal development among university teachers (Capelo et al., 2022), so that they can serve as good role models.

We argue that, a single “positive” trait, a stable temperament profile, and even a creative (SCT) character profile might be necessary but definitely not sufficient for optimal health and well-being and for improving the human condition. It is rather the coherence of temperament and character that determines positive health outcomes. In order to face these unprecendent times, nursing education might need to focus on helping students to, besides professional skills, develop health-related abilities, such as self-acceptance and spiritual acceptance, by promoting self-awareness. Thus, current curricula for nursing education might need to be developed to not only prepare students to take care of their future patients; but also to develop resilient-related skills. The same goes for their future workplaces, public health policy needs to emphasize a learning working climate that support nurses to continue developing their own health and personality, which will also make them good role models for their patients.

### Funding

Nothing to declare.

## CRediT authorship contribution statement

**Danilo Garcia:** Writing – review & editing, Writing – original draft, Supervision, Project administration, Methodology, Investigation, Funding acquisition, Formal analysis, Conceptualization. **Maryam Kazemitabar:** Writing – review & editing, Methodology, Formal analysis, Data curation. **Elina Björk:** Writing – review & editing, Investigation. **Thiago Medeiros da Costa Daniele:** Writing – review & editing. **Marko Mihailovic:** Writing – review & editing. **Kevin M. Cloninger:** Writing – review & editing. **Mirna Albuquerque Frota:** Writing – review & editing. **C.Robert Cloninger:** Writing – review & editing, Supervision, Methodology, Conceptualization.

## Declaration of competing interest

The authors declare that they have no competing interests.

## Appendix Supplementary materials

Image, application 1
