# Supplementary material for: Nursing students' personality (Temperament and Character), burnout symptoms, and health and well-being
Source: Int J Nurs Stud Adv. 2024 May 6;6:100206. doi: 10.1016/j.ijnsa.2024.100206 (PMC11129095; doi:10.1016/j.ijnsa.2024.100206)
Supplement: Supplementary file 1 [file mmc1.pdf]

SUPPLEMENTARY MATERIAL

Nursing Students' Personality (Temperament and Character), Burnout Symptoms, and Health and Well-Being

Danilo Garcia [1, 2, 3, 4, 5*@], Maryam Kazemitabar[6*], Elina Björk[2, 3], Thiago Medeiros da Costa Daniele[3, 7], Marko Mihailovic[2, 3], Kevin M. Cloninger[2, 8], Mirna Albuquerque Frota[7], C. Robert Cloninger[2, 8]

[1]Department of Behavioral Sciences and Learning, Linköping University, Linköping, Sweden

[2]Lab for Biopsychosocial Personality Research (BPS-PR) Lab, International Network for Well-Being

[3]Promotion of Health and Innovation (PHI) Lab, International Network for Well-Being

[4]Centre for Ethics, Law and Mental Health (CELAM), University of Gothenburg, Gothenburg, Sweden

[5]Department of Psychology, University of Gothenburg, Gothenburg, Sweden

[6]Yale School of Medicine, Yale University, New Haven, Connecticut, USA

[7]Programa de Pós-graduação em Saúde Coletiva, University of Fortaleza (UNIFOR), Ceará, Brazil

[8]Anthropedia Foundation, St. Louis, Missouri, USA

* These authors contributed equally to this work. [@]Correspondence regarding this article should be addressed to D. Garcia, danilo.garcia@icloud.com.

The Biopsychosocial Model of Personality

Cloninger's biopsychosocial model of personality has its basis in the development of the human brain through evolution, which gave birth to three types of memory and learning systems: the *procedural system*, responsible for behavioral conditioning of habits and skills; the *propositional system*, responsible for intentionality including executive self-control for attaining goals, semantic learning of facts, and abstract symbolization; and the *episodic system*, responsible for self-awareness including a person's own life narrative and identity (Cloninger, 2009, 2004).

On this theoretical framework, human personality is the dynamic organization, within an individual, of psychobiological systems that modulate adaptation to a changing environment (Cloninger, 2004; Cloninger et al., 1993). Hence, personality includes metacognitive patterns and systems that regulate different aspects that involve being human: cognition, emotion and mood, personal impulse control, and social relations; including relating to, and thinking about oneself, other people, and the world as a whole (Cloninger, 2009, 2004). Consequently, our personality influences and is reflected in the different domains of our life, such as sexuality, physicality, emotionality, sociality, cognition, and spirituality. Evolution is, after all, a complex adaptive process in which multiple genetic and environmental events are constantly interacting, shifting the balance of reproductive fitness from situation to situation and time to time (Wright, 1982). Certainly, the genetic influences on personality and other human characteristics show extensive gene–gene and gene–environmental interactions (Cloninger, 2004; Cloninger et al., 2019; Josefsson et al., 2013b; Keltikangas-Jarvinen et al., 2009, 2004).

The procedural memory and learning system regulates different emotional responses such as joy, sadness, anger, fear, disgust, and ambition, that is, human temperament. According to Cloninger's model of personality there are four temperament traits in modern humans: (1) *Novelty*

Seeking, associated with the neurotransmitter dopamine, is expressed as frequent exploration of new unfamiliar places or situations, quick loss of temper, impulsive decision making, and active avoidance of monotony; (2) *Harm Avoidance*, associated with the neurotransmitter serotonin, is expressed as fear of uncertainty, shyness of strangers, quick fatigability, and pessimistic worry of future problems; (3) *Reward Dependence*, associated with the neurotransmitter noradrenaline, is the tendency to respond intensively to reward expressed as sentimentality, social attachment, and dependence of approval of others; and (4) *Persistence*, also associated to the brain's noradrenergic system, is the tendency to persevere despite fatigue or frustration, overachieving, and perfectionism. The temperament traits are useful to predict disorders and destructive behaviors, but not sufficient to predict who will develop a disorder or maladaptive behaviors (Cloninger et al., 1993)—substance abuse, for example, is associated with high levels of Novelty Seeking, but not all individuals who are high in Novelty Seeking develop substance abuse problems.

The second system of learning and memory, the propositional system, is present in primates and helps the individual to be self-directed and cooperative in a social environment. The third system, the episodic system, exists only among humans and stands for humans' capacity for self-awareness, which allows introspection and recollection of autobiographical memories (Cloninger, 2004). Ergo, the second and third systems are responsible for the presence of human character, which can be defined as individual differences in values, goals and self-conscious emotions (e.g., hope, love, and faith) or what people make of themselves intentionally (Cloninger, 2004). That is, while temperament refers to the way we are born (i.e., our emotional predispositions), character traits describe individual differences in our self–object relationships, which begin with parental attachments in infancy, then self–object differentiation in toddlers, and continue to mature in a stepwise manner throughout life (Cloninger, 2004). The three character traits are: *Self-*

Directedness (1), which refers to self-determination, being able to control, regulate, and adapt behavior in accordance to own goals and values, to be self-sufficient, self-acceptant, responsible, dependable, and effective; *Cooperativeness* (2) accounts for individual differences in acceptance of and identification with other people, tolerance, helpfulness, and empathy; and *Self-Transcendence* (3) which refers to individual differences in selflessness or self-forgetfulness, patience, spirituality, and identification with something bigger than the self that gives meaning to one's existence, such as humanity, nature, God, or the universe (Cloninger, 2004; Cloninger et al., 1993).

Importantly, these three systems of learning and memory can be dissociated functionally from one another, but normally interact jointly so that habits, goals, and values can be integrated (Cloninger, 2004; Cloninger and Cloninger, 2011; Garcia et al., 2019a, 2019c, 2019b). Indeed, in three recent molecular genetic studies (Zwir et al., 2021, 2020a, 2020b) using three culturally diverse samples (Finland, Germany, and South Korea), we found that variants of clusters of genes, rather than single genes, are associated with personality—these genes do not only operate independently; but are organized as sets of particular Single-Nucleotide Polymorphisms (SNPs) that co-occur in subgroups of individuals. The SNPs sets were each comprised of SNPs in many coding and noncoding genes that are distributed throughout the genome. Thus, each gene can be expected to affect many traits and many genes affect each trait because evolutionary selection operates on whole organisms, not individual genes or traits (Zwir et al., 2022, 2021, 2020a, 2020b). In addition, as originally proposed by Cloninger, we found that the genes that encode variability in human temperament are enriched in highly conserved molecular pathways, which are activated in experimental animals by stress reactivity and associative conditioning (i.e., the procedural brain system). In contrast, the genes encoding for human character involve specialized bipolar neurons

that are functionally connected to neocortical regions in brain circuits that support saliency detection, resolution of emotional conflicts, and social cooperation for mutual benefit in great apes and humans (i.e., the prepositional brain system). The character genes also involved regions of late-myelinating neocortex in frontal, parietal, and temporal regions found only in humans that are associated with the emergence of human capacities for self-awareness, insight (i.e., immediate, accurate, and deep intuitive understanding), creative imagination, altruism, and autobiographical memory (i.e., the episodic brain system) (Zwir et al., 2022, 2021, 2020a, 2020b).

In other words, personality is a complex adaptive system of patterns of relationships between temperament structure, character structure, and histories of behavioral conditioning and insight (social) learning, that can be measured as a biopsychosocial model of personality (Figure 1 in the main text). Seeing personality as a dynamic complex adaptive system entails a nonlinear approach that is person-centered and in which an individual is not only adapting to the environment, but also to the traits within the person (Bergman et al., 2003; Bergman and Magnusson, 1997; Bergman and Wångby, 2014; Cloninger et al., 1997; Cloninger and Zwir, 2022, 2018)—that is, the notion of the individual as whole system unit which is best studied by analyzing profiles. Hence, these nonlinear dynamics might provide further information to understand academic burnout and lack of good health and well-being.

Joint Personality (Temperament-Character) Networks

Accordingly, to the notion of personality as a complex adaptive system, we found three nearly separate joint personality networks of people with different combinations of temperament and character: *Creative/Reliable*, *Organized/Reliable*, *Emotional/Unreliable* (Zwir et al., 2021). There was marked complexity within each network in temperament-character relations. People in the Creative/Reliable network were low in Novelty Seeking (i.e., deliberate, thrifty, and orderly), low

in Harm Avoidance (i.e., optimistic, confident, outgoing, and vigorous), high in Reward Dependence (i.e., sentimental, friendly, and approval seeking), and high in Persistence (i.e., determined) in combination with a mature character development (i.e., high in all three character traits), thus, leading to high levels of well-being and good health. Individuals in the Organized/Reliable network had the same temperament configurations as those in the Creative/Reliable network and were high in both Self-Directedness and Cooperativeness, but not in Self-Transcendence, which makes them healthy, but vulnerable to existential crises. Individuals in the Emotional/Unreliable network had the highest level of ill-being and the lowest level of well-being, which is logical since they were high in Novelty Seeking (i.e., impulsive and extravagant), high in Harm Avoidance (i.e., pessimistic, fearful, shy, and fatigable), and high in Reward Dependence (i.e., sentimental and friendly) in combination with an immature character development (i.e., low in all three character traits). This means that they may frequently have approach-avoidance conflicts, rejection sensitivity, disorganized attachments, and different biopsychosocial health issues. Individuals in the Creative/Reliable network had the highest levels of well-being, but also had a slightly higher risk of ill-being than those in the Organized/Reliable network.

A recent cross-sectional set of studies of American, Portuguese, and Bulgarian populations have replicated these results (Garcia et al., 2022; Moreira et al., 2023, 2022, 2021). Although such cross-sectional studies confound effects of aging with differences among birth cohorts, the results are consistent with extensive biopsychosocial evidence that women are more prosocial and healthier on average, particularly in Cooperativeness and Self-Transcendence. That being said, character maturity develops with age; thus, a Creative/Reliable joint personality network and high Self-Transcendence are relatively unlikely to be common in a young sample of nursing students.

Nevertheless, traits as Cooperativeness and Reward Dependence should be relatively high among nursing students, who are mostly women and have opted for a caring profession (Mihailovic et al., 2022).

Latent Profile Analysis (LPA): Temperament Profiles

The LPA using the four temperament dimensions as the profiling variable resulted in different models with distinct numbers of profiles. As indicated in Table S1, we tested the fit indices for four temperament models. Although Model 4 had the lowest AIC and SABIC and the highest entropy, profile 2 in this model had 2.2% of the population—profiles smaller than 5% of the whole sample are usually considered spurious and non-replicable (Hipp and Bauer, 2006). Of the remaining models, Model 2 had the lowest BIC, but Model 3 had three distinct profiles with sufficient percentage of nursing students in each profile. In Model 3, about 23.5% of the nursing students were clustered in temperament profile 1 and scored low in Novelty Seeking (n), high in Harm Avoidance (H), high in Reward Dependence (R), and low in Persistence (p). About 68.5% were clustered in temperament profile 2 and scored low in Novelty Seeking (n), low in Harm Avoidance (h), high in Reward Dependence (R), and high in Persistence (P). Finally, 8.4% were clustered in temperament profile 3 and scored low in Novelty Seeking (n), high in Harm Avoidance (H), high in Reward Dependence (R), and high in Persistence (P) (see Figure S1). Hence, we chose Model 3 as the best model to describe how the different complex dynamics between temperament dimensions were expressed as temperament profiles. Next, we proceeded to validate and understand this further by investigating differences in temperament within and between individuals with the three distinct temperament profiles in Model 3.

Table S1. Fit indices for the four Temperament models extracted using LPA.

Model	AIC	BIC	SABIC	Entropy	VLMRT	LMRT	BLRT
**1**	1746.119	1773.194	1747.843				
**2**	1728.977	1772.975	1731.779	0.454	0.1704	0.1808	0.0000
**3**	1724.999	1785.920	1728.879	0.658	0.4629	0.4733	0.2273
**4**	1715.288	1793.131	1720.246	0.767	0.0251	0.0278	0.0000

Note. The number of the model also indicates the number of profiles in that specific model. Green indicates the chosen model.

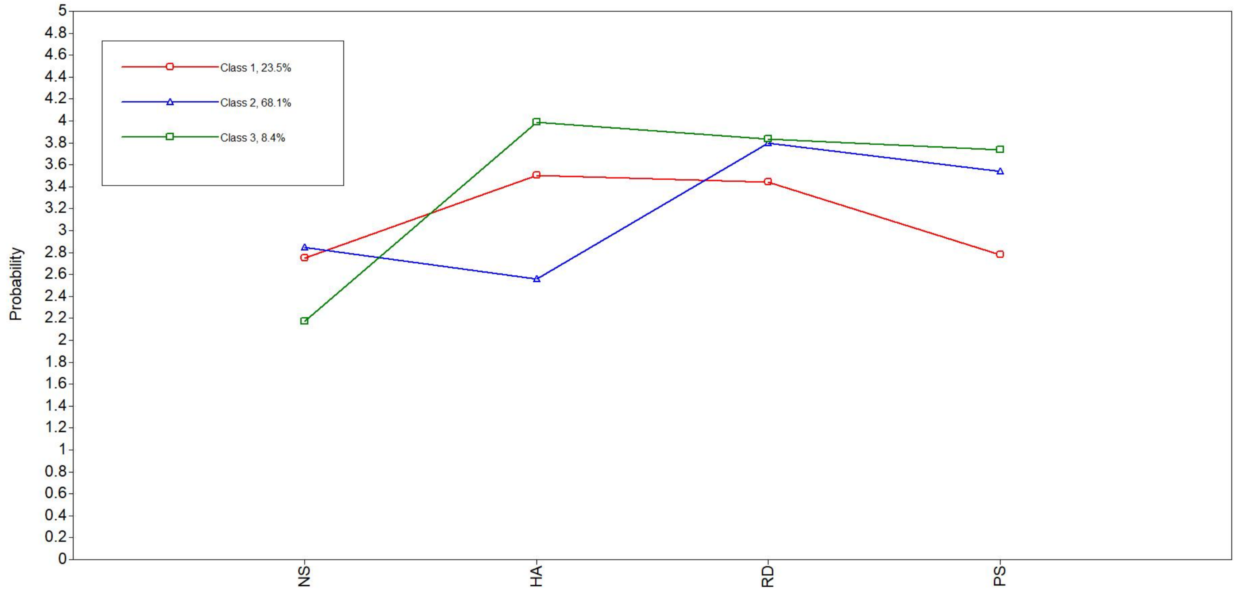

Figure S1. Probability plot showing the three temperament profiles of the most valid model calculated using Latent Profile Analysis (LPA).

Note: NS = Novelty Seeking, HA = Harm Avoidance, RD = Reward Dependence, PS = Persistence. Class = Profile.

Differences in Temperament Dimensions within Temperament Profiles

The differences in temperament dimensions within each temperament profile were measured separately using repeated measures ANOVA. Significant effects were further investigated using post hoc tests with Bonferroni correction. For temperament profile 1, the test of within-subject effects with Greenhouse-Geisser correction was significant ($F_{(2.36,141.39)} = 46.54$, $p < .001$, partial Eta squared = 0.44). The post hoc test showed that all mean differences within temperament

dimension were significant except for that between Harm Avoidance and Reward Dependence and between Novelty Seeking and Persistence ($p > .05$). The test within-subjects effect for temperament profile 2 with Greenhouse-Geisser correction indicated significant differences as well ($F_{(2.87, 447.84)} = 162.80$, $p < .001$, partial Eta squared = 0.51). The post hoc test showed that all mean differences within temperament dimension were significant ($p < .01$). Finally, the test of within-subject effects for temperament profile 3 also suggested significant differences within this profile with a Greenhouse-Geisser correction test ($F_{(2.24, 35.84)} = 68.90$, $p < .001$, partial Eta squared = 0.81). The post hoc test showed that differences between Harm Avoidance and Reward Dependence; Harm Avoidance and Persistence; and Reward Dependence and Persistence were not significant ($p > .05$). In other words, within this profile, only Novelty Seeking was significantly lower compared to the other traits which where all equally high. Hence, the differences in temperament dimensions within the temperament profiles were more accentuated for nursing students in profile 2, moderately in profile 1 and to a lesser degree in profile 3. For instance, for individuals in temperament profile 3, this means that they are equally worried and shy (high Harm Avoidance) as warm and approval-seeking (high Reward Dependence) and as perfectionistic (high Persistence), but they are extremely rigid, orderly, and conventional (low Novelty Seeking). This specific difference within the individual might lead to wanting to keep order and being conventional but being extremely worried and shy about hurting other people's feelings at the same time that the individual is extremely persistent in all these three behaviors. On the other hand, nursing students in profile 2 were more optimistic and outgoing (low Harm Avoidance) than rigid and conventional (low Novelty Seeking). Even though they were still low in Novelty Seeking, they worry less and are optimistic and warm and approval-seeking.

Differences in Temperament Dimensions between Temperament Profiles

Using a one-way MANOVA, we found significant differences in temperament dimensions between the three temperament profiles (Wilks' Lambda = 0.28, $F_{(8, 424)} = 46.86$, $p < .001$, partial Eta squared = 0.47). Pairwise comparison, using a Bonferroni correction to the alpha level, indicated that all differences were significant except for profiles 1 and 2 concerning Novelty Seeking ($p > .05$), for profiles 2 and 3 with regard to Reward Dependence and Persistence ($p > .05$), and for profiles 1 and 3 with regard to Reward Dependence ($p > .05$). In other words, although all three temperament profiles were low in Novelty Seeking, profile 3 was slightly lower. Harm Avoidance was significantly different between all profiles—with higher levels among nursing students in profile 3 and lowest levels in profile 2. Reward Dependence was high and relatively equal in all three profiles. Last but not least, Persistence was equally high among nursing students in profiles 2 and 3 but higher than nursing students in profile 1 (see Figure S2).

The Temperament Profiles

The LPA and validation analyses conducted showed that the whole nursing student population (all three profiles) is low in Novelty Seeking (n) and high in Reward Dependence (R). That is, they are orderly, warm, and sympathetic and are also rigid and approval-seeking. Most nursing students, those in profile 2 (68.5%), reported being optimistic, outgoing, and stable (i.e., low in Harm Avoidance). Likewise, most of them reported being hard-working, determined, and perfectionistic (i.e., high Persistence), but those in profiles 2 and 3 (76.9% in total) reported higher levels in this temperament dimension.

The nursing students in temperament profile 1 might be described as inhibited (nH), rejection-sensitive (HR), and traditional (nR). Consequently, they avoid doing anything that exposes them to the risk of danger, rejection, criticism, and testing new ways of doing things.

Nevertheless, they have much social warmth (R) and are likely to be careful and dutiful in carrying out responsibilities assigned to them (n). However, they have difficulty initiating anything new because of their inhibitions rooted in their tendency to pragmatism and underachievement (p) and their fears of rejection, criticism, loss, and change (HR). Hence, we labeled temperament profile 1 as pessimistic (nHRp) (Cloninger, 2004). The nursing students in temperament profile 3 were almost as "cautious" and "warm" (nR) as those in temperament profile 1. However, the analyses showed that nursing students in profile 3 reported higher levels of both Harm Avoidance (H) and Persistence than those in profile 1. The largest difference, however, was that nursing students in profile 3 were extremely more persistent (P) and showed less differences within temperament dimensions. In other words, nursing students in profile 3 are more persistent in their tendencies towards being inhibited (nH), rejection-sensitive (HR), and traditional (nR) (Cloninger, 2004). Hence, we refer to this profile as "painstaking" (nHRP). It is noteworthy to mention that profile 3 had a smaller number of people.

Finally, the nursing students in temperament profile 2 might be described as "reliable" (nhRP). Individuals with this temperament profile are stable (nh), warmly sociable (hR), traditional (nR), and hard-working (P). Hence, it is highly likely that they can be trusted to carry out what they are expected to do in a predictable and traditional manner and to develop a mature character (Cloninger, 2004).

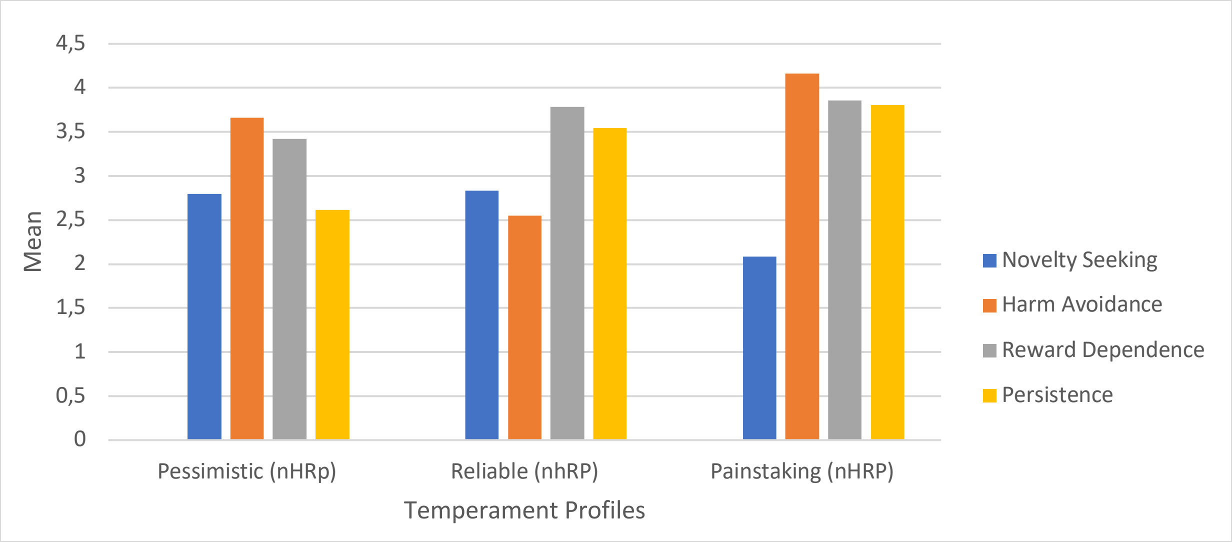

Figure S2. Mean differences in temperament dimension between and within nursing students with the distinct three temperament profiles from Model 3.

Note. n = low Novelty Seeking, H = high Harm Avoidance, h = low Harm Avoidance, R = high Reward Dependence, P = high Persistence, p = low persistence.

Differences in Burnout Symptoms and Health and Well-Being between Nursing Students with Distinct Temperament Profiles

We investigated differences in burnout symptoms and health and well-being between nursing students with the three distinct temperament profiles using a MANOVA. The results showed that nursing students were significantly different with regard to burnout symptoms and health and well-being (Wilks' Lambda = 0.69, $F_{(8, 424)} = 10.73$, $p < .001$, partial Eta squared = 0.17). A post hoc test with a Bonferroni correction showed that students in the pessimistic (nHRp) temperament profile and students in the painstaking (nHRP) temperament profile did not differ in Emotional Exhaustion, Cynicism, and health and well-being ($p > .05$); and that students in the reliable (nhRP) temperament profile and students in the painstaking (nHRP) temperament profile did not differ in Academic Efficacy ($p > .05$). In other words students in the pessimistic (nHRp) and the painstaking (nHRP) profiles reported equally high levels of Emotional Exhaustion and Cynicism and equally low levels of health and well-being; while being equally low in Novelty Seeking (n), equally high in Harm Avoidance (H) and Reward Dependence (R), but differing in Persistence (p/P). Therefore,

it is plausible to link both high and low levels of Persistence to feeling emotionally drained by one's studies, feeling doubt regarding the significance of one's studies, and ill-being. In other words, being perseverant and perfectionistic (P) on one side and being easily discouraged and underachiever (p) on the other, will both lead to burnout and ill-being if the individual is, at the same time, rigid and orderly (n), has the tendency to worry and being pessimistic (H), and is sympathetic and approval-seeking (R). On the other hand, high Persistence was linked to high levels of self-efficacy regarding one's studies (i.e., Academic Efficacy), regardless levels of high Harm Avoidance, when Novelty Seeking was low and Reward Dependence was (nR). Indeed, nursing students in the reliable (nhRP) temperament profile did not differ in this specific burnout symptom compared to those in the painstaiking (nHRP) one. That being said, nursing students in the reliable (nhRP) temperament profile reported, as expected, significantly less burnout symptoms and better health and well-being than those nursing students in any of the other two temperament profiles. See Figure S3 for the details.

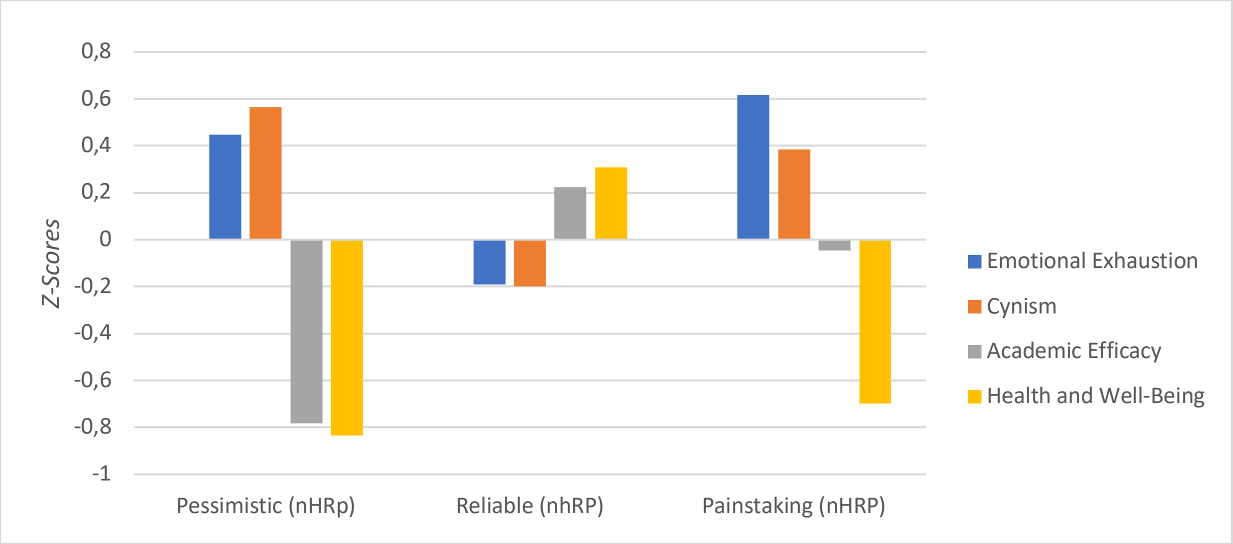

Figure S3. Mean (*z-scores*) differences in burnout symptoms and health and well-being between nursing students with the three distinct temperament profiles in Model 3.

Note. n = low Novelty Seeking, H = high Harm Avoidance, h = low Harm Avoidance, R = high Reward Dependence, P = high Persistence, p = low persistence.

Latent Profile Analysis (LPA): Character Profiles

The LPA using the three character dimensions as the profiling variable resulted in different models with distinct numbers of profiles. As indicated in Table S2, we tested the fit indices for four character models. Although Model 4 had the most optimum AIC and SABIC, this model had one profile with a very low number of nursing students (2.3%), which was insufficient to be considered as a separate profile (Hipp & Bauer, 2006). Model 2 had the lowest BIC and highest entropy but both profiles extracted in this model were similar: high Cooperativeness, low Self-Transcendence, and moderate Self-Directedness. As detailed in the probability plot (Figure S4), Model 3 was comprised of profile 1 with about 11.1% of the nursing students who were low in Self-Directedness (s), low in Cooperativeness (c), and low in Self-Transcendence (t); profile 2 with about 56.2% of the nursing students who were high in Self-Directedness (S), high in Cooperativeness (C), and low in Self-Transcendence (t); and profile 3 with about 32.6% of the nursing students who were high in Self-Directedness (S), high in Cooperativeness (C) and high in Self-Transcendence (T). Hence, we chose Model 3 as the best model to describe how the different complex dynamics between character dimensions were expressed as character profiles. Next, we proceeded to validate and understand this further by investigating differences in character within and between the three character profiles in Model 3.

Table S2. Fit indices for the four character models extracted using LPA

Models	AIC	BIC	SABIC	Entropy	VLMRT	LMRT	BLRT
**1**	1248.162	1268.469	1249.456				
**2**	1220.438	1254.283	1222.594	0.736	0.0049	0.0060	0.0000
**3**	1216.280	1263.663	1219.298	0.634	0.4657	0.4827	0.0500
**4**	1213.193	1274.114	1217.074	0.725	0.0471	0.0520	0.1500

Note. The number of the model also indicates the number of profiles in that specific model. Green indicates the chosen model.

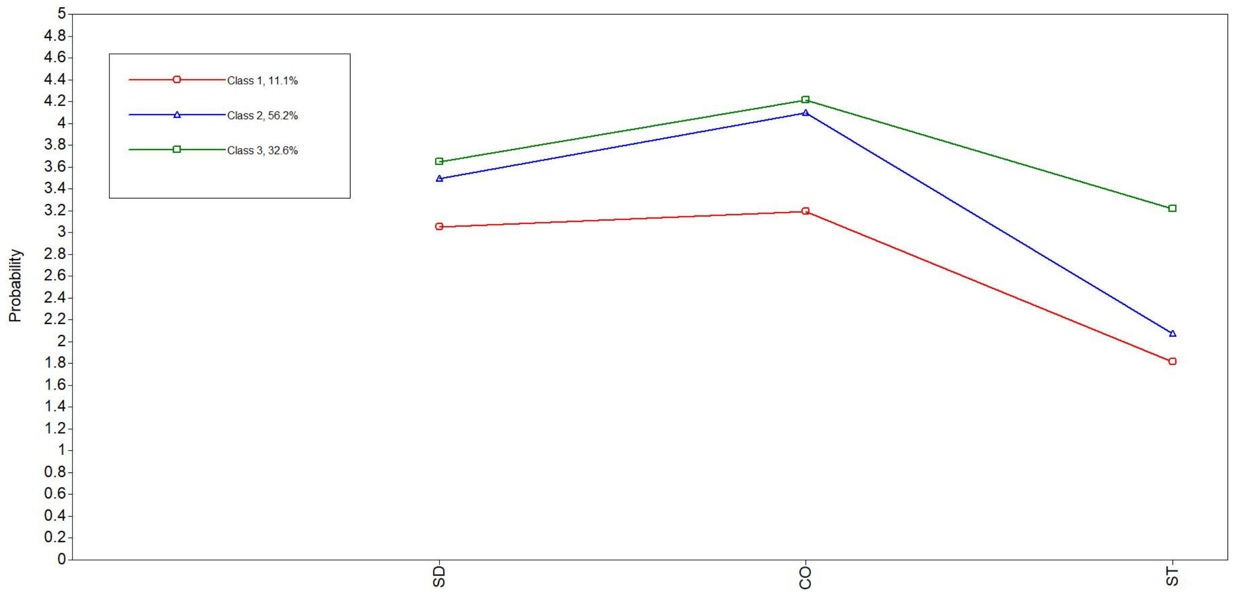

Figure S4. Probability plot showing the three character profiles of the most valid model calculated using Latent Profile Analysis (LPA).

Note: SD = Self-Directedness, CO = Cooperativeness, ST = Self-Transcendence. Class = Profile.

Differences in Character Dimensions within Character Profiles

We found significant differences in character dimensions within character profiles using repeated measures ANOVA, one ANOVA for each profile. Significant effects were further investigated using post hoc tests with Bonferroni correction. The test of within-subject effects for character profile 1 using Greenhouse-Geisser correction showed that there were significant differences within character dimensions ($F_{(1.98, 39.49)} = 47.67$, $p < .001$, partial Eta squared = 0.70). The post hoc test showed that individuals in profile 1 reported equal levels of Self-Directedness and Cooperativeness ($p > .05$) and that they reported significantly lower levels of Self-Transcendence than any of the other two character traits ($p < .001$). The test of within-subject effects using a Greenhouse-Geisser correction for character profile 2 also showed significant mean differences within character dimensions in this profile ($F_{(1.87, 240.58)} = 559.234$, $p < .001$, partial Eta squared = 0.81). The post hoc test showed that all character dimensions differed from each other within character profile 2 ($p < .001$). In other words, nursing students located in character profile 2

reported higher levels of Cooperativeness than Self-Directedness and Self-transcendence, whilst Self-Transcendence was the lowest of all character traits within this profile. Finally, we then tested within-subject effect differences using a Greenhouse-Geisser test showed that the character dimensions among the nursing students in this profile differed significantly from each other ($F_{(1.95, 128.89)} = 57.316$, $p < .001$, partial Eta squared = 0.47). The post hoc test showed that all mean differences were significant ($p < .001$), although lesser between Self-Directedness and Self-Transcendence ($p < .01$). That is, nursing students located in character profile 3 reported higher levels of Cooperativeness than Self-Directedness and Self-transcendence, but almost equal levels of Self-Directedness and Self-Transcendence.

Differences in Character Dimensions between Character Profiles

Using a one-way MANOVA, we found significant differences in character dimensions (Wilks' Lambda = 0.32, $F_{(6, 426)} = 54.80$, $p < .001$, partial Eta squared = 0.44) between the three character profiles. A post hoc test using Bonferroni correction to the alpha level indicated that all differences in character dimensions between profiles were significant except for Self-Directedness and Cooperativeness between character profiles 2 and 3 ($p > .05$). In other words, nursing students in profile 1 reported lower levels of Self-Directedness, Cooperativeness, and Self-Transcendence compared to those in the other two profiles. Moreover, students in profiles 2 and 3 had equally high levels of Self-Directedness and Cooperativeness, but those in profile 3 reported higher levels of Self-Transcendence. Figure S5 depicts the differences within and between profiles in the three character dimensions.

The Character Profiles

The LPA and validation analyses showed that a majority (i.e., profiles 1 and 2 or about 67.3%) of the nursing student population is low in Self-Transcendence. In other words, they are

individualistic, skeptical, conventional, and cynical (Cloninger, 2004). A majority is relatively high in both Self-Directedness and Cooperativeness (i.e., profiles 2 and 3 or about 88.8%). That is, they are responsible, resourceful, self-acceptant, have a sense of purpose (i.e., high Self-Directedness) and are tolerant, empathetic, forgiving, helpful, and principled (i.e., high Cooperativeness) (Cloninger, 2004).

The nursing students in character profile 1 feel very often victimized and helpless due to low Self-Directedness and low Cooperativeness (sc), show very poor judgment due to low Self-Directedness and low Cooperativeness (st) and are distrustful due to low Cooperativeness and low Self-Transcendence (ct). In other words, they experience the world from an outlook of separateness, which leads to fear, excessive desire, and false pride or self-reproach. Hence, we labeled character profile 1 as "apathetic" (sct) (Cloninger, 2004). Indeed, individuals with this type of character report the lowest levels of overall well-being and health and experience unhealthy emotions such as anxiety and alienation and have high rates of mental and physical disorders (Cloninger, 2004).

The nursing students in character profile 2 might often be perceived as mature leaders due to high levels of both Self-Directedness and Cooperativeness (SC), logical due to high Self-Directedness and low Self-Transcendece (St), and conventional due to high Cooperativeness and low Self-transcendence (Ct). Thus, we labeled this character profile "organized" (SCt) (Cloninger, 2004). Individuals with an organized character profile are, most of the time, happy and healthy, and seldom need health care. However, when they face difficult existential challenges, such as severe illness or death, they often lack the necessary outlook of unity and connectedness needed to be resilient through such situations because they are low in Self-Transcendence (t).

Lastly, the nursing students with character profile 3 might be described as constructive, tend to keep things in perspective when faced with challenges (high Self-Directedness = S), enjoy helping others, are compassioned (high Cooperativeness = C), and seek to grow in awareness of things that go beyond human existence (high Self-Transcendence = T) (Cloninger, 2004). We labeled this profile as "creative" (SCT) (Cloninger, 2004). Individuals with a creative (SCT) character profile consistently report the highest levels of well-being, healthy longevity, optimal cardiovascular health, including a healthy lifestyle as well as reduced risk for chronic diseases (Cloninger, 2004). The levels of Self-Directedness and Self-Transcendence in this sample, however, were relatively average. Then again, the nursing students were relatively young and are therefore expected to increase in character maturity—in Scandinavia as in the rest of the world people's Self-Directedness and Cooperativeness increase from the age 25 to 35, but contrary to the rest of the world, in Scandinavia people decreases in Self-Transcendence (Josefsson et al., 2013a).

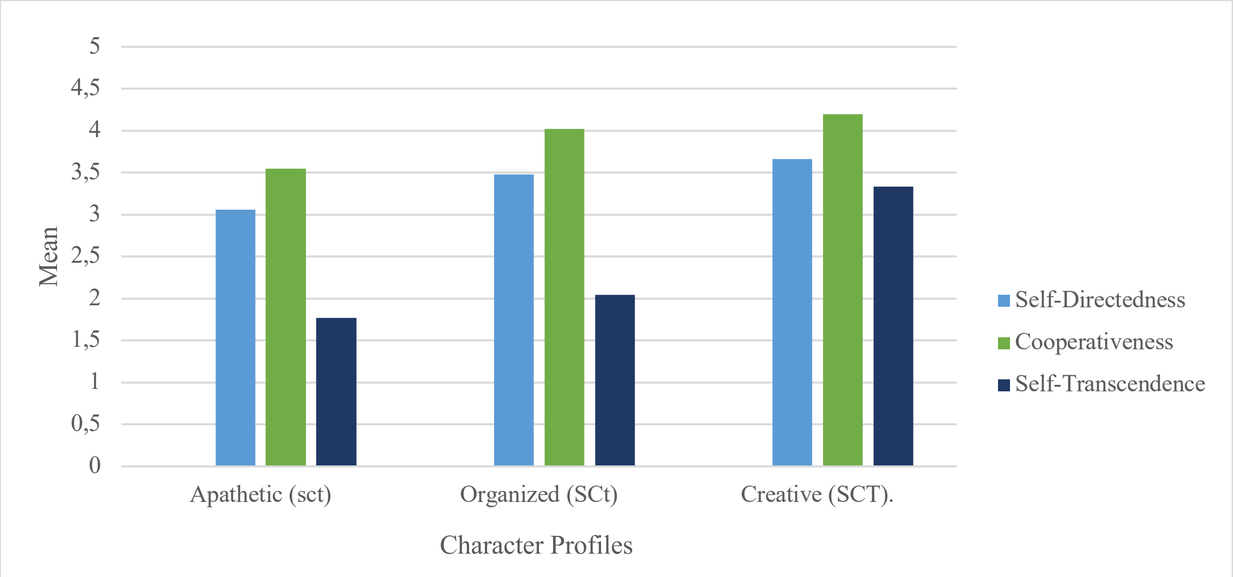

Figure S5. Mean differences in character dimension between and within nursing students with the three distinctic Character profiles in Model 3.

Note. S = high Self-Directedness, s = low self-directedness, C = high Cooperativeness, c = low cooperativeness, T = high Self-Transcendence, t = low self-transcendence.

Differences in Burnout Symptoms and Health and Well-Being between Nursing Students with Distinct Character Profiles

We investigated mean differences in burnout symptoms and health and well-being between nursing students with the three distinct character profiles using a MANOVA, which revealed significant differences (Wilks' Lambda = 0.87, $F_{(8, 424)} = 3.75$, $p < .001$, partial Eta squared = 0.07). The test between-subject effects showed that Emotional Exhaustion and Cynicism differences in all three character profiles were nonsignificant ($p > .05$). Moreover, a post hoc test with Bonferroni correction showed that differences in Academic Efficacy were higher among nursing students in the creative (SCT) character profile compared to those in any of the other two profiles. Students in the apathetic (sct) character profile and students in the organized (SCt) profile did not differ in either Academic Efficacy or health and well-being. Lastly, students in the organized (SCt) character profile and students in the creative (SCT) character profile did not differ in health and well-being ($p > .05$), but those in the creative (SCT) profile reported significantly better health and well-being than those in the apathetic (sct) profile (p < .01). In other words suggesting that when Self-Directedness and Cooperativeness are high (i.e., nursing students in both the organized and creative profile), the individual can achieve high Academic Efficacy and good health and well-being; but it is only when Self-Transcendence is high at the same time (i.e., creative profile) that levels in these two outcomes are higher than those reported by students in the apathetic (sct) character profile. See Figure S6 for the details.

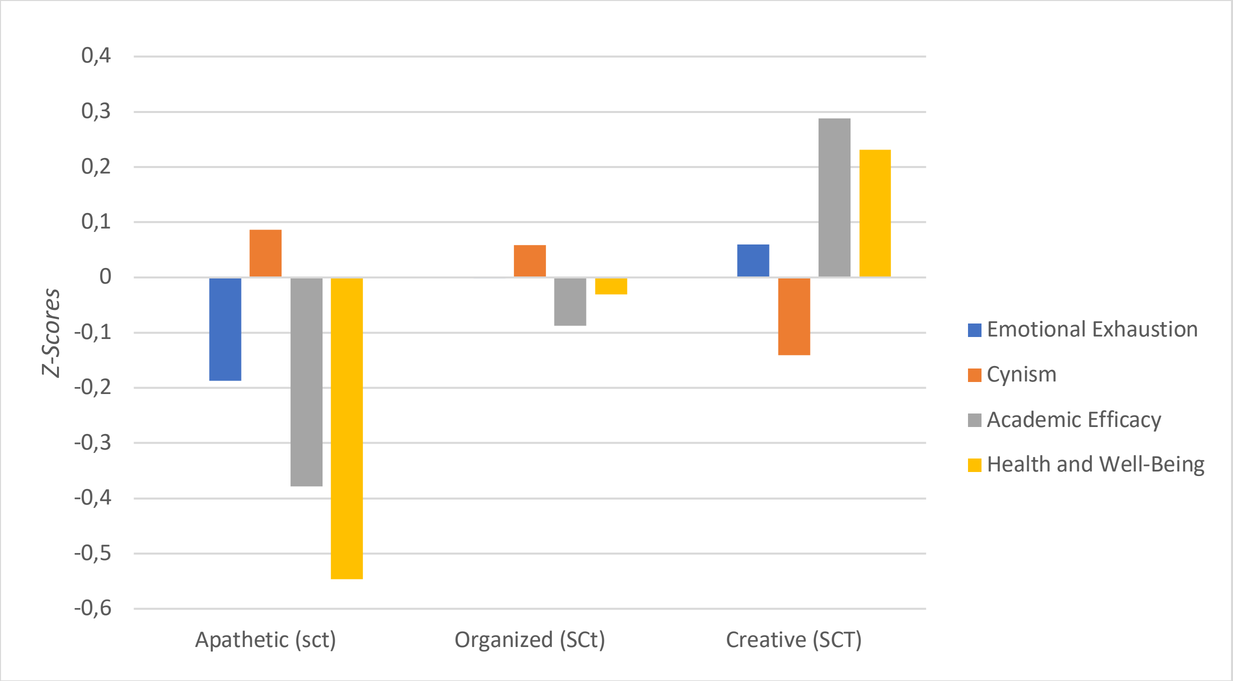

Figure S6. Mean (*z-scores*) differences in burnout symptoms and health and well-being between nursing students with the three distinct temperament profiles in Model 3.

Note. S = high Self-Directedness, s = low self-directedness, C = high Cooperativeness, c = low cooperativeness, T = high Self-Transcendence, t = low self-transcendence.

Latent Class Analysis (LCA): Joint Personality (Temperamet-Character) Networks

The LCA for the joint personality (temperament-character) networks resulted in different models with distinct numbers of profiles. We tested the fit indices for four joint personality network models (see Table S3; the model number also stands for the number of profiles in that specific model) and found that Model 2 had the lowest AIC, BIC, and SABIC and the highest entropy. In Figure S7, we show the probability plots in Model 2 with two networks, which were evaluated as the model that best fit the data. About 40.7% of the nursing students were highly likely to belong to joint personality network 1 and about 59.3% to joint personality network 2. Table S4 displays the number and percentage of nursing students with distinct temperament and character profiles clustered in each joint personality network and also the probability that nursing students with specific temperament profiles and character profiles belong to each joint personality network.

The majority of the nursing students in the joint personality network 1 had a pessimistic (nHRp) temperament profile (58.8%) or a painstaking (nHRP) temperament profile (25.0%). Importantly, all nursing students in the sample who had a painstaking (nHRP) temperament profile belonged to this joint personality network, despite that the probability for students with the pessimistic (nHRp) and the reliable (nhRP) temperament profiles were relatively equal (i.e., 41.2% and 39.2%, respectively). Indeed, as much as 97.3% of the nursing students in joint personality network 2 had a reliable (nhRP) temperament profile and none of them had a painstaking (nHRP) temperament profile. In other words, all nursing students with painstaking (nHRP) temperament profile and the majority of those with a pessimistic (nHRp) temperament profile were allocated to joint personality network 1; while almost all nursing students, but 2.7%, allocated to joint personality network 2 had a reliable (nhRP) temperament profile.

Moreover, about 60.3% of the students in joint personality network 1 had an organized (SCt) character profile. 16.2% had an apathetic (sct) character profile, and 23.5% had a creative (SCT) character profile. In the joint personality network 2, 59.3% had an organized (SCt) character profile, 34.0% had a creative (SCT) character profile (probability = 51.8%), and 6.7% had an apathetic (sct) character profile. That is, as much as 76% of the nursing students with a creative (SCT) character profile and 68% of those with an organized (SCt) character profile were allocated to the joint personality network 2. However, this also means that about one-third of the nursing students with an organized (SCt) character profile and about ¼ of those with a creative (SCT) character profile were allocated to joint personality network 1. Moreover, about half of the nursing students with an apathetic (sct) character profile were allocated to each joint personality network.

That being said, except for four individuals, all nursing students in joint personality network 2 had a reliable (nhRP) temperament profile, thus suggesting a stable temperament for all

students allocated to this network, including the few who had an apathetic (sct) character profile—80% of nursing students with an apathetic (sct) character profile within joint personality network 2 had a functional temperament profile, that is, reliable (nhRP). Conversely, most nursing students in joint personality network 1 had a type of pessimistic (nHR) temperament profile with either high or low Persistence (P/p)— 83% of the students with an organized (SCt) character profile and 81% of the students with a creative (SCT) character profile within joint personality network 1 had a dysfunctional temperament profile, that is, either pessimistic (nHRp) or painstaking (nHRP). Hence, in general, joint personality network 2 consisted of individuals with a reliable (nhRP) temperament profile and those with a creative (SCT) or an organized (SCt) profile. Next, we proceeded to validate and understand this further by investigating differences in temperament and character dimensions within and between the joint personality networks.

Table S3. Fit indices for the four joint personality (temperament-character) network models extracted using Latent Class Analysis (LCA).

Models	AIC	BIC	SABIC	Entropy	VLMRT	LMRT	BLRT
**1**	729.423	742.961	730.285				
**2**	707.988	738.449	709.928	0.517	0.0000	0.0000	0.0000
**3**	715.053	762.436	718.071	0.516	0.1000	0.1069	0.1034
**4**	725.053	789.359	729.149	0.488	0.0000	0.0000	1.0000

Note. The number of the model also indicates the number of networks in that specific model. Green indicates the chosen model.

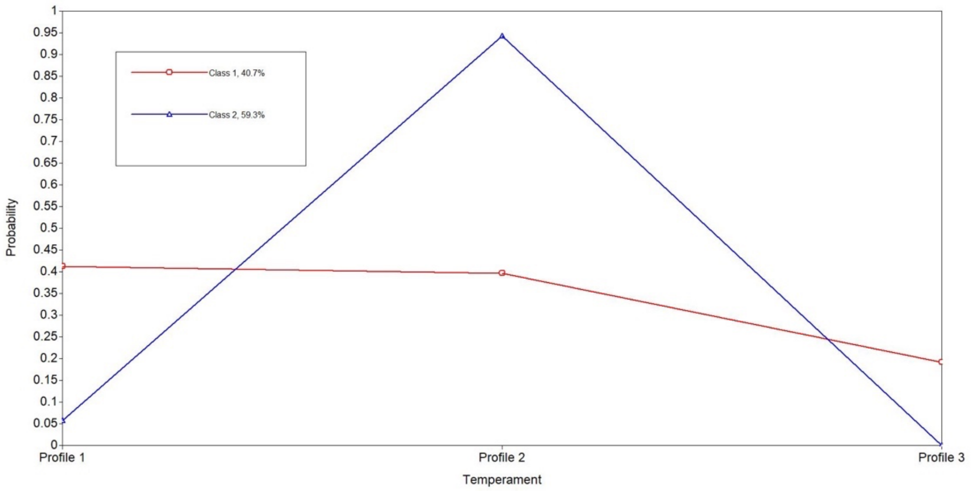

A.

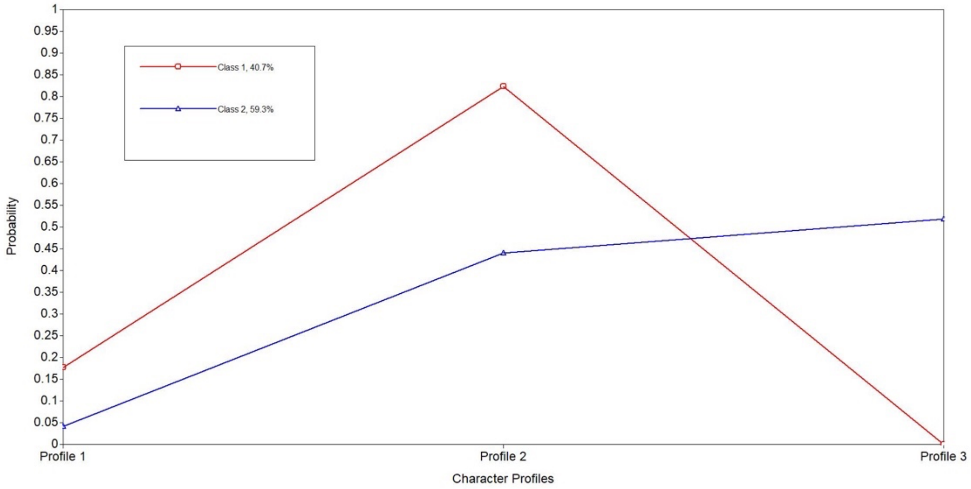

B.

Figure S7. Probability plot showing the three temperament profiles (A) and the three character profiles of the most valid joint personality (temperament-character) model calculated using Latent Class Analysis (LCA).

Note. For temperament profiles (A): profile 1 = pessimistic (nHRp), profile 2 = reliable (nhRP), and profile 3 = painstaking (nHRP). For character profiles (B): profile 1 = apathetic (sct), profile 2 = organized (SCt), and profile 3 = creative (SCT).

n = low Novelty Seeking, H = high Harm Avoidance, h = low Harm Avoidance, R = high Reward Dependence, P = high Persistence, p = low persistence, S = high Self-Directedness, s = low self-directedness, C = high Cooperativeness, c = low cooperativeness, T = high Self-Transcendence, t = low self-transcendence.

Table S4. Percentage of nursing students with different temperament and character profiles clustered in each of the joint personality (temperament-character) networks.

	Profiles	Joint Personality Network 1		Joint Personality Network 2		Total
		n	%	*n*	%	*N* (%)
**Temperament Profiles**	Pessimistic (nHRp)	40	58.8%	4	2.7%	44 (20.2%)
	Reliable (nhRP)	11	16.2%	146	97.3%	157 (72%)
	Painstaking (nHRP)	17	25.0%	0	0%	17 (7.8%)
**Total**		68	100%	150	100%	218 (100%)
**Character Profiles**	Apathetic (sct)	11	16.2%	10	6.7%	21 (9.6%)
	Organized (SCt)	41	60.3%	89	59.3%	130 (59.6%)
	Creative (SCT)	16	23.5%	51	34.0%	67 (30.7%)
**Total**		68	100%	150	100%	218 (100%)

Note. n = low Novelty Seeking, H = high Harm Avoidance, h = low Harm Avoidance, R = high Reward Dependence, P = high Persistence, p = low persistence, S = high Self-Directedness, s = low self-directedness, C = high Cooperativeness, c = low cooperativeness, T = high Self-Transcendence, t = low self-transcendence.

Differences in Temperament and Character Dimensions within Joint Personality (Temperament-Character) Networks

We investigated the differences in temperament and character dimensions within each joint personality network using two separate repeated measures ANOVA. Significant effects were further investigated using post hoc tests with Bonferroni correction. For the joint personality network 1, the test of within-subject effects with Greenhouse-Geisser correction was significant ($F_{(4.85, 325.03)} = 56.76$, $p < .001$, *partial Eta squared* = .46). The post hoc test showed that, within this joint personality network, Novelty Seeking was significantly lower than all other personality dimensions but Persistence and Self-Transcendence. Regarding Harm Avoidance, it was equally high as Reward Dependence and Cooperativeness, but significantly higher than any of the other

personality dimensions. Besides being equally high as Harm Avoidance and significantly lower than Cooperativeness, Reward Dependence was significantly higher than all the rest of the personality dimensions. For Persistence, besides being as low as Novelty Seeking, it was also as low Self-Directedness and significantly lower than all other dimensions. In turn, Self-Directedness was significantly lower than Harm Avoidance, Reward Dependence, and Cooperativeness; equally low as Persistence, but significantly higher than Novelty Seeking and Self-Transcendence. Besides being as high as Harm Avoidance, Cooperativeness was significantly higher than the rest of the personality dimensions. Finally, besides being equally low as Novelty Seeking, Self-Transcendence was significantly lower than all other dimensions.

The test within-subjects effect for the joint personality network 2 with Greenhouse-Geisser correction indicated significant differences as well ($F_{(5.12,\ 305.07)} = 178.73$, $p < .001$, *partial Eta squared* = .55). The post hoc test showed that Novelty Seeking was significantly higher than Harm Avoidance and Self-Transcendence and lower than all the rest of the personality dimensions. Harm Avoidance, in turn, was significantly lower than all dimensions, but Self-Transcendence. Reward Dependence was significantly lower than Cooperativeness, equally high as Self-Directedness, and significantly higher than any of the other personality dimensions. For Persistence, besides being significantly lower than Reward Dependence and Cooperativeness, it was equally high as Self-Directedness and significantly higher than Novelty Seeking, Harm Avoidance, and Self-Transcendence. In turn, Self-Directedness was equally high as Persistence and Reward Dependence, significantly lower than Cooperativeness and significantly higher than Novelty Seeking, Harm Avoidance, and Self-Transcendence. Cooperativeness was significantly higher than all personality dimensions. Finally, besides being equally low as Novelty Seeking, Self-Transcendence was significantly lower than all other dimensions (see Figure S8).

Differences in Temperament and Character Dimensions between Joint Personality (Temperament-Character) Networks

Using a one-way MANOVA, we found significant differences in temperament and character dimensions between the two joint personality networks in Model 2 (*Wilks' Lambda* = .58, $F_{(7, 210)}$ = 21.65, $p < .001$, *partial Eta squared* = .42). Pairwise comparison, using a Bonferroni correction to the alpha level, indicated that nursing students in joint personality network 1 scored significantly lower in Novelty Seeking, significantly higher in Harm Avoidance, significantly lower in Persistence, significantly lower in Self-Directedness, equally high in Reward Dependence and Cooperativeness, and equally low in Self-Transcendence compared to nursing students with the joint personality network 2. Figure S8 displays the mean differences in temperament and character dimensions within and between the two extracted joint personality networks.

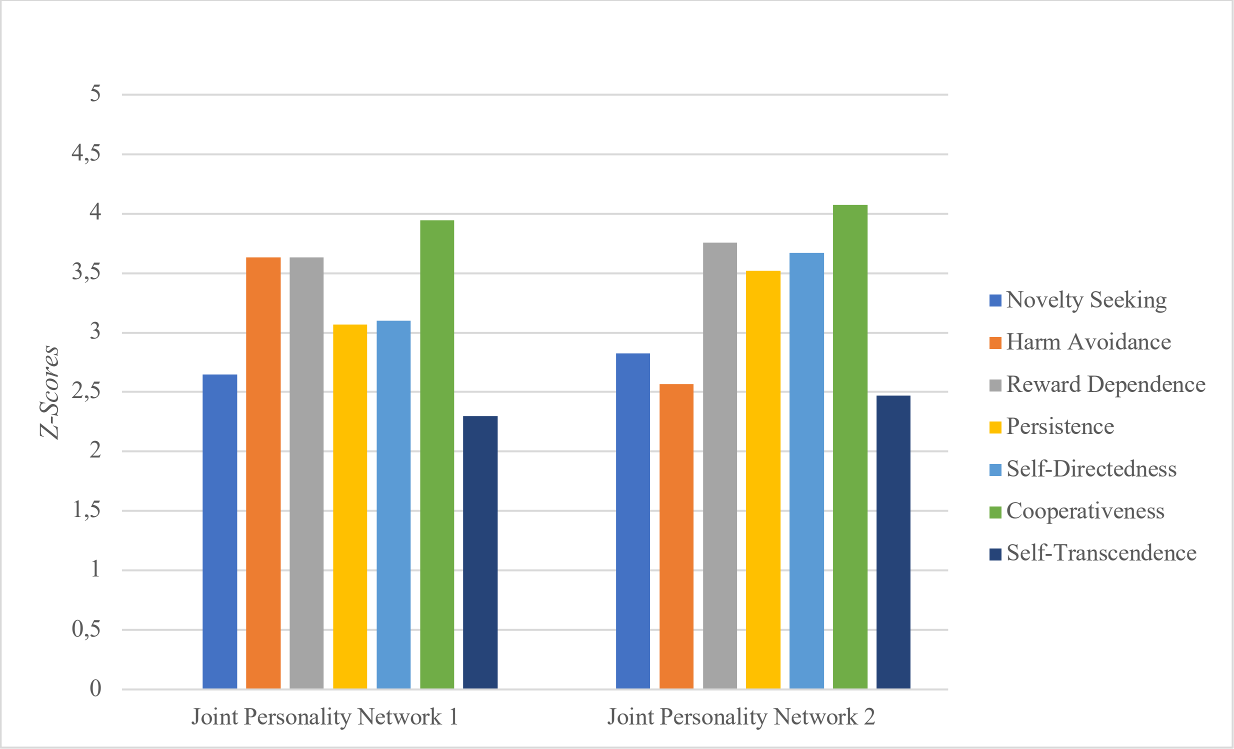

Figure S8. Mean differences in temperament and character dimensions between and within joint personality networks in Model 2.

References

Bergman, L.R., Magnusson, D., 1997. A person-oriented approach in research on developmental psychopathology. Dev Psychopathol 9. https://doi.org/10.1017/S095457949700206X

Bergman, L.R., Magnusson, D., Khouri, B.M., 2003. Studying individual development in an interindividual context: A person-oriented approach. Psychology Press. https://doi.org/10.4324/9781410606822

Bergman, L.R., Wångby, M., 2014. The person-oriented approach: A short theoretical and practical guide. Eesti Haridusteaduste Ajakiri 2. https://doi.org/10.12697/eha.2014.21.02b

Cloninger, C.R., 2009. Evolution of human brain functions: the functional structure of human consciousness. Australian and New Zealand Journal of Psychiatry 43, 994–1006. https://doi.org/10.3109/00048670903270506

Cloninger, C.R., 2004. Feeling good: The science of well-being. Oxford University Press.

Cloninger, C.R., Cloninger, K.M., 2011. Person-centered Therapeutics. The International Journal of Person Centered Medicine 1, 43–52. https://doi.org/10.5750/ijpcm.v1i1.21

Cloninger, C.R., Cloninger, K.M., Zwir, I., Keltikangas-Järvinen, L., 2019. The complex genetics and biology of human temperament: a review of traditional concepts in relation to new molecular findings. Transl Psychiatry 9, 290. https://doi.org/10.1038/s41398-019-0621-4

Cloninger, C.R., Svrakic, D.M., Przybeck, T.R., 1993. A psychobiological model of temperament and character. Arch Gen Psychiatry 50.

Cloninger, C.R., Svrakic, N.M., Svrakic, D.M., 1997. Role of personality self-organization in development of mental order and disorder. Dev Psychopathol 9. https://doi.org/10.1017/S095457949700148X

Cloninger, C.R., Zwir, I., 2022. Genetics of Human Character and Temperament, in: ELS. Wiley, pp. 1–20. https://doi.org/10.1002/9780470015902.a0029415

Cloninger, C.R., Zwir, I., 2018. What is the natural measurement unit of temperament: Single traits or profiles? Philosophical Transactions B 373. https://doi.org/10.1098/rstb.2017.0163

Garcia, D., Cloninger, K.M., Lester, N., Cloninger, C.R., 2019a. The future of personality research and applications – some latest findings, in: Garcia, D., Archer, T., Kostrzewa, R.M. (Eds.), Personality and Brain Disorders: Associations and Interventions. Springer, Cham. https://doi.org/10.1007/978-3-319-90065-0

Garcia, D., Cloninger, K.M., Sikström, S., Anckarsäter, H., Cloninger, C.R., 2019b. A ternary model of personality: Temperament, character, and identity, in: Sikström, S., Garcia, D. (Eds.), Statistical Semantics: Methods and Applications. Springer, Cham.

Garcia, D., Kazemitabar, M., Stoyanov, D., Cloninger, C.R., 2022. Differences in subjective well-being among individuals with distinct joint personality (temperament-character) in a Bulgarian sample. PeerJ 10. https://doi.org/10.7717/peerj.13956

Garcia, D., Lester, N., Lucchese, F., Cloninger, K.M., Cloninger, C.R., 2019c. Personality and the brain: Person-centered approaches, in: Garcia, D., Archer, T., Kostrzewa, R.M. (Eds.), Personality and Brain Disorders: Associations and Interventions. Springer. https://doi.org/10.1007/978-3-319-90065-0_1

Hipp, J.R., Bauer, D.J., 2006. Local solutions in the estimation of growth mixture models. Psychol Methods 11. https://doi.org/10.1037/1082-989X.11.1.36

Josefsson, K., Jokela, M., Cloninger, C.R., Hintsanen, M., Salo, J., Hintsa, T., Keltikangas-Jarvinen, L., 2013a. Maturity and change in personality: Developmental trends of temperament and character in adulthood. Dev Psychopathol 25. https://doi.org/10.1017/S0954579413000126

Josefsson, K., Jokela, M., Hintsanen, M., Cloninger, C.R., Pilkki-Råback, L., Merjonen, P., Hutri-Kähönen, N., Keltikangas-Järvinen, L., 2013b. Parental care-giving and home environment predicting offspring's temperament and character traits after 18 years. Psychiatry Res 209. https://doi.org/10.1016/j.psychres.2013.01.007

Keltikangas-Jarvinen, L., Pulkki-Raback, L., Elovainio, M., Raitakari, O.T., Viikari, J., Lehtimaki, T., 2009. DRD2 C32806T modifies the effect of child-rearing environment on adulthood novelty seeking. American Journal of Medical Genetics. Part B, Neuropsychiatric Genetics 150B.

Keltikangas-Jarvinen, L., Raikkonen, K., Ekelund, J., Peltonen, L., 2004. Nature and nurture in novelty seeking. Mol Psychiatry 9.

Mihailovic, M., Garcia, D., Amato, C., Lindskär, E., Rosenberg, P., Björk, E., Lester, N., Cloninger, K.M., Cloninger, C.R., 2022. The personality of newly graduated and employed nurses: Temperament and character profiles of Swedish nurses. Int J Nurs Stud Adv 4, 100058. https://doi.org/10.1016/j.ijnsa.2021.100058

Moreira, P.A.S., Inman, R.A., Cloninger, C.R., 2023. Disentangling the personality pathways to well-being. Sci Rep 13, 3353. https://doi.org/10.1038/s41598-023-29642-5

Moreira, P.A.S., Inman, R.A., Cloninger, C.R., 2022. Virtues in action are related to the integration of both temperament and character: Comparing the VIA classification of virtues and Cloninger's biopsychosocial model of personality. J Posit Psychol 17, 858–875. https://doi.org/10.1080/17439760.2021.1975158

Moreira, P.A.S., Inman, R.A., Cloninger, C.R., 2021. Personality Networks and Emotional and Behavioral Problems: Integrating Temperament and Character Using Latent Profile and Latent Class Analyses. Child Psychiatry Hum Dev 52, 856–868. https://doi.org/10.1007/s10578-020-01063-9

Wright, S., 1982. The shifting balance theory and macroevolution. Annu Rev Genet 16.

Zwir, I., Arnedo, J., Del-Val, C., Pulkki-Råback, L., Konte, B., Yang, S.S., Romero-Zaliz, R., Hintsanen, M., Cloninger, K.M., Garcia, D., Svrakic, D.M., Rozsa, S., Martinez, M., Lyytikäinen, L.-P., Giegling, I., Kähönen, M., Hernandez-Cuervo, H., Seppälä, I., Raitoharju, E., de Erausquin, G.A., Raitakari, O., Rujescu, D., Postolache, T.T., Sung, J., Keltikangas-Järvinen, L., Lehtimäki, T., Cloninger, C.R., 2020a. Uncovering the complex genetics of human temperament. Mol Psychiatry 25, 2275–2294. https://doi.org/10.1038/s41380-018-0264-5

Zwir, I., Arnedo, J., Del-Val, C., Pulkki-Råback, L., Konte, B., Yang, S.S., Romero-Zaliz, R., Hintsanen, M., Cloninger, K.M., Garcia, D., Svrakic, D.M., Rozsa, S., Martinez, M., Lyytikäinen, L.-P., Giegling, I., Kähönen, M., Hernandez-Cuervo, H., Seppälä, I., Raitoharju, E., de Erausquin, G.A., Raitakari, O., Rujescu, D., Postolache, T.T., Sung, J., Keltikangas-Järvinen, L., Lehtimäki, T., Cloninger, C.R., 2020b. Uncovering the complex genetics of human character. Mol Psychiatry 25, 2295–2312. https://doi.org/10.1038/s41380-018-0263-6

Zwir, I., Del-Val, C., Arnedo, J., Pulkki-Råback, L., Konte, B., Yang, S.S., Romero-Zaliz, R., Hintsanen, M., Cloninger, K.M., Garcia, D., Svrakic, D.M., Lester, N., Rozsa, S., Mesa, A., Lyytikäinen, L.-P., Giegling, I., Kähönen, M., Martinez, M., Seppälä, I., Raitoharju, E., de Erausquin, G.A., Mamah, D., Raitakari, O., Rujescu, D., Postolache, T.T., Gu, C.C., Sung, J., Lehtimäki, T., Keltikangas-Järvinen, L., Cloninger, C.R., 2021. Three genetic–environmental networks for human personality. Mol Psychiatry 26, 3858–3875. https://doi.org/10.1038/s41380-019-0579-x

Zwir, I., Del-Val, C., Hintsanen, M., Cloninger, K.M., Romero-Zaliz, R., Mesa, A., Arnedo, J., Salas, R., Poblete, G.F., Raitoharju, E., Raitakari, O., Keltikangas-Järvinen, L., de Erausquin, G.A., Tattersall, I., Lehtimäki, T., Cloninger, C.R., 2022. Evolution of genetic networks for human creativity. Mol Psychiatry 27, 354–376. https://doi.org/10.1038/s41380-021-01097-y
